# Conductive MOFs with Photophysical Properties: Applications and Thin-Film Fabrication

**DOI:** 10.1007/s40820-020-00470-w

**Published:** 2020-06-19

**Authors:** Zeyu Zhuang, Dingxin Liu

**Affiliations:** Skate Key Laboratory of Optoelectronic Materials and Technologies, Nanotechnology Research Center, School of Materials Science and Engineering, Sun Yat-Sen University, Guangzhou, 510275 People’s Republic of China

**Keywords:** Metal–organic frameworks, Photoconductivity, Photoluminescence, Thin films

## Abstract

Metal–organic frameworks (MOFs) are a class of hybrid materials with many promising applications. In recent years, lots of investigations have been oriented toward applications of MOFs in electronic and photoelectronic devices. While many high-quality reviews have focused on synthesis and mechanisms of electrically conductive MOFs, few of them focus on their photophysical properties. Herein, we provide an in-depth review on photoconductive and photoluminescent properties of conductive MOFs together with their corresponding applications in solar cells, luminescent sensing, light emitting, and so forth. For integration of MOFs with practical devices, recent advances in fabrication of photoactive MOF thin films are also summarized.

## Highlights

- An overview on photophysical properties of conductive metal–organic frameworks (MOFs) including photoconductivity and photoluminescence is provided.
- Miscellaneous applications of MOFs with photophysical properties are discussed.
- Recent advances in integration of photoactive MOFs with practical devices are summarized.

## Graphical abstract

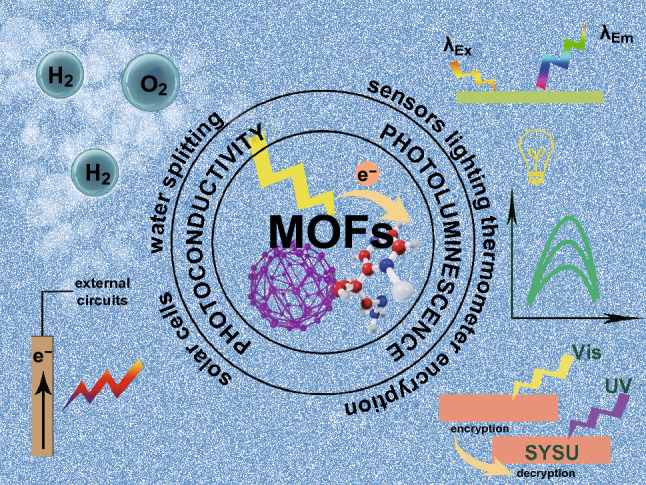

## Introduction

As a collective class of crystalline materials containing metal nodes connected by organic ligands, metal–organic frameworks (MOFs) have attracted much attention [1]. The high porosity, stability and exceptional topological and compositional tunability make MOFs applicable in many fields such as gas storage, separation [2], catalysis [3] and ionic transport [4]. In recent years, more and more conductive MOFs have been designed and synthesized with their electrical conduction nature widely discussed. Conductive MOFs have been demonstrated as promising materials to improve technologies such as energy conversion and storage, electrochemical capture and release, battery systems, chemical sensing, and catalysis [5]. Especially, under irradiation of laser, conductive MOFs generally exhibit surprising reactions such as the change of electrical conductivity and light emission effect [6]. As is known to all, light energy has played a more and more significant role in modern society due to its renewability and eco-friendliness. The effective utilization of light energy will help alleviate energy crisis. These laser-induced photophysical properties of conductive MOFs expand their applications in light harvesting, analyte sensing and so on and provide another possible way to utilize the light energy [7]. Furthermore, with the help of advances in fabrication of MOF thin films, it is enabled to integrate functional MOFs with electronic and optoelectronic devices.

While many excellent reviews have focused on the synthesis, mechanisms, and miscellaneous applications of conductive MOFs [8–11], few of them focus on their photophysical properties (i.e., their responses under irradiation of laser). Therefore, in this review, we will discuss in detail the photophysical properties of conductive MOFs. Specifically, we provide an in-depth review on the photoconductive and photoluminescent properties of MOFs as well as their corresponding applications in solar cells, luminescent sensors, lighting devices, and so forth. In addition, for integration in practical devices, MOFs need to be prepared in forms of thin films, so in the last section we will discuss recent advances in deposition of MOF thin films that exhibit exceptional photophysical properties and hold a bright prospect in electronic and optoelectronic fields.

## Photoconductivity

### Photoconductive MOFs

The band gap theory accounts for conductive or insulating properties of many MOFs. For MOFs with large band gap between the valence band (VB) and conduction band (CB), it is usually hard to realize charge transfer and hence electrical conductivity. Upon irradiation at wavelengths exceeding the band gap, electrons can be excited from the VB to CB, which arouse electron–hole separation with positive holes created in the VB and negative electrons in the CB. Based on the above band gap mechanism, lowering the band gap is a promising strategy for synthesis of photoconductive MOFs, which exhibit increased electrical current under illumination and can possibly function as photoactive electrodes for many optoelectrical applications such as water splitting and solar cells. In general, the band gap of MOFs with electron donor–acceptor pairs is relatively narrow. In MOFs of donor–acceptor architecture, electrons are released by the electron donor and the electron acceptor further promotes the charge transfer by enhancing electron–hole separation and inhibiting electron–hole recombination. Therefore, it is a promising strategy to synthesize photoconductive MOFs through donor–acceptor architecture, which usually involves photoactive organic compounds. Besides photoconductivity based on the organic moieties, attempts have also been made to explore the effects of inorganic building unit on photoconductive properties of MOFs.

#### Photoconductivity Based on Organic Moieties

*Electron*-*accepting ligands* As metal centers in MOFs tend to emit electrons due to their reduction property, they usually serve as electron donors. Therefore, electron-accepting organic ligands are typically involved in construction of donor–acceptor architecture for photoluminescent MOFs. With suitable band gap, electrons will be generated and transferred from the metal center to ligand upon irradiation at some wavelengths.

**Fig. 1:**
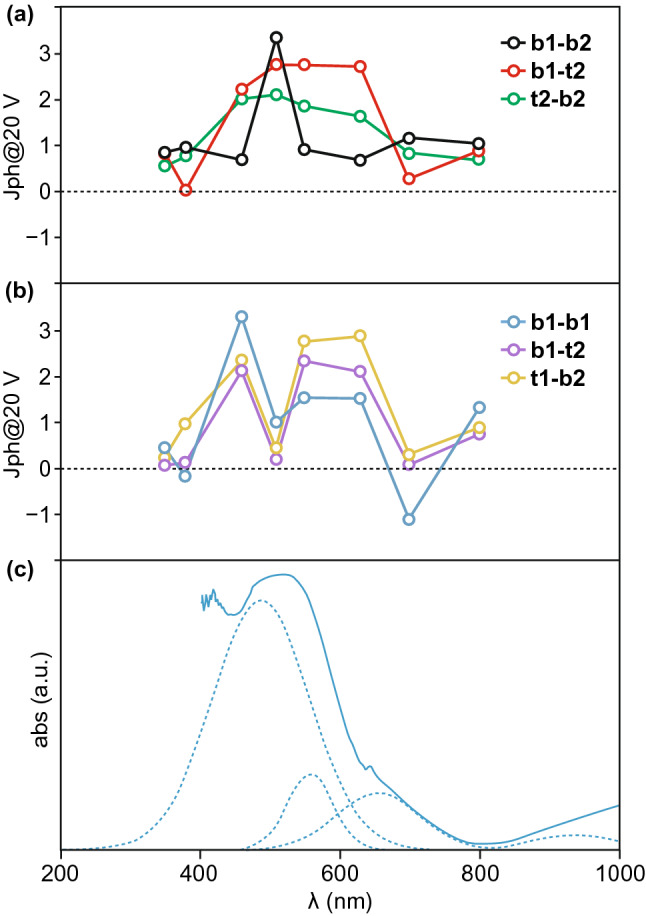
**a**, **b** Photoresponse intensities at 20 V (different combinations of b1, b2 and t1, t2 stand for different crystal orientations). **c** Electronic absorption spectrum of MOF-CoNDI-py-2. Reprinted with permission from Ref. [12]. Copyright 2017, Springer Nature

1,4,5,8-Naphthalene diimides (NDIs) are a class of organic compounds with excellent semiconductive and optical properties. On basis of this, the photoactive response of MOF-CoNDI-py-2, featuring Co(II), *N*,*N*′-bis(4-pyridyl)-1,4,5,8-naphthalene diimide (NDI-py) and terephthalic acid (TpA), was observed [12]. Upon irradiation, a charge transfer from the metal center to the π-acceptor NDI-py occurred, which promoted hole transport through the Co–TpA direction and electron transport through the NDI-py direction. As shown in Fig. 1, the as-synthesized MOF exhibited anisotropic photoconductivity and the highest photoresponse intensities (*J*_ph_) obtained coincided with the charge transfer band. Interestingly, in this case, a photoresistive–photoresponsive dual behavior was observed. While mostly the current increased upon illumination, at negative bias sometimes the current decreased under illumination at some wavelengths. This special photoresistance phenomenon could be ascribed to the metal centers as charge trap sites in their oxidation state which may impede the charge transfer.

Another typical example is the 3D framework {[Cu^I^Cu_2_^II^(DCTP)_2_]NO_3_·1.5DMF}_n_ (DCTP = 4′-(3,5-dicarboxyphenyl)-4,2′:6′,4′’-terpyridine) with a narrow band gap of 2.1 eV [13]. Upon irradiation, electrons jumped into the CB and holes were generated in the VB. Local density of states (LDOS) and partial density of sates (PDOS) analysis revealed that the valance-band maximum (VBM) was dominated by Cu 3d orbitals and the conduction-band minimum (CBM) mainly consisted of 2p orbitals of C and N of the ligand. Thereby, the excited electrons transferred from Cu to neighboring C and N atoms. Notably, for this photoconductive MOF, the CBM was higher than H^+^/H_2_ energy level and the VBM was lower than O_2_/H_2_O level, enabling the production of H_2_ and O_2_ with this MOF under irradiation.

**Fig. 2:**
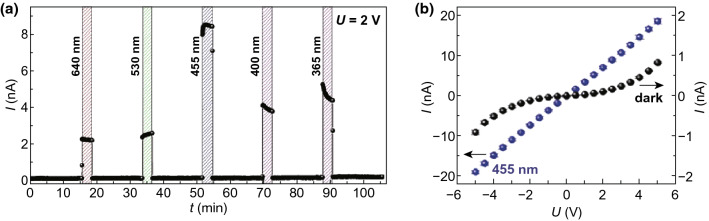
**a** DC current *I* at a voltage of 2 V under irradiations with light of 640, 530, 455, 400, and 365 nm wavelength. **b** Current–voltage curve of the sample in the dark (black spheres) and under irradiation with 455 nm (blue spheres). Reprinted with permission from Ref. [14]. Copyright 2019, Wiley. (Color figure online)

*Electron*-*donating ligands* Not all ligands serve as electron acceptors in photoconductive MOFs. For some electron-donating ligands, guest molecules are usually required to form donor–acceptor pairs. Porphyrins, for example, are excellent electron donors with delocalized *π*-systems. Recently, Liu et al. have conducted research to compare the photoconductivity of Cu(BDPC) and Zn(TPP) SURMOFs with embedded C_60_ fullerene and found that the physical properties of both SURMOFs were considerably distinct although they shared the very similar lattice constants and pore sizes [14]. While C_60_-loaded Cu(BDPC) responded to light irradiation slightly with their conductivity almost unaffected by irradiation of light of various wavelengths, the opposite was true for C_60_@Zn(TPP), which was ascribed to the different linkers in them as Cu(BDPC) possessed phenyl-based linkers and Zn(TPP) porphyrinic linkers. As shown in Fig. 2a, applying 2 V to the C_60_@Zn(TPP) sample, the current increased from 0.11 in the dark to 9 nA upon irradiation of photon wavelength at 455 nm (blue light). Figure 2b shows that the current increased with voltage roughly exponentially in the dark, whereas for light of 455 nm, the current was proportional to the voltage, revealing almost ideal ohmic conduction behavior with a conductivity of 1.3 × 10^−7^ S cm^−1^, corresponding to a conductivity increase upon illumination by 2 orders of magnitude. The photoconductivity of C_60_@Zn(TPP) was attributed to the interaction of electron-donor porphyrin linkers and electron-acceptor C_60_ guest molecules. Upon irradiation, the Soret band of porphyrin was activated, enabling the generation of electron–hole pairs, and at the same time, C_60_ significantly improved the separation and transfer of electron–hole pairs, restraining their recombination and the electron back-transfer. Furthermore, it is possible to modify the active components, porphyrin and fullerene, without changing the crystal structure. C_60_-COOH@Zn(DAP) with a different porphyrin linker (DAP = [10,20-bis(4-carboxyphenyl)5,15-diazaporphyrinato]zinc(II)) was thus synthesized and showed similar photoconductance properties.

**Fig. 3:**
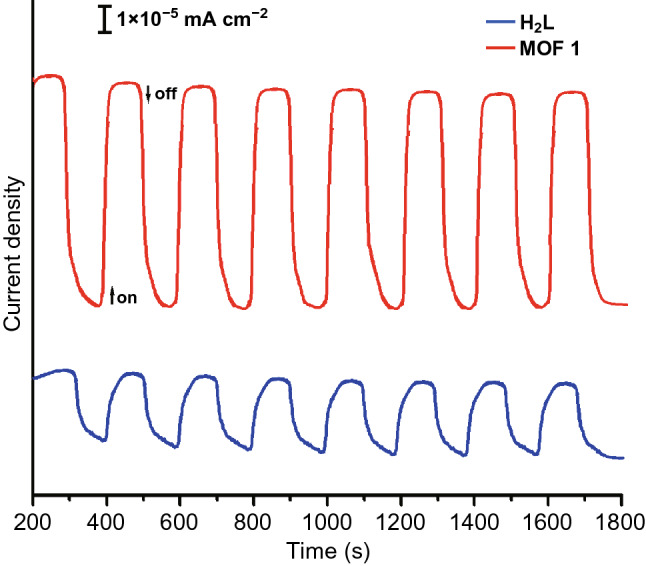
Comparison of photocurrent time plots of ZnL(DPE)(H_2_O)·H_2_O and free H_2_L ligand. Reprinted with permission from Ref. [15]. Copyright 2016, Elsevier

It has been revealed that the delocalized π electrons can effectively decrease the band gap of MOFs and hence promote photoconductive properties. A research on 4-(4-oxopyridin-1(4H)-yl)phthalic acid (H_2_L) and three H_2_L-based MOFs ZnL(DPE)(H_2_O)·H_2_O (DPE = (E)-1,2-di(pyridine-4-yl)ethene), CdL(H_2_O)_2_ and CdL was conducted [15]. Even though the three as-synthesized MOFs shared the same L^2−^ ligand, the band gap of the first MOF was much lower than that of either the other two or the free H_2_L ligand, which was ascribed to the presence of DPE ligand in the first MOF. DPE ligand as N-donor was a planar molecule full of π electrons over the large conjugated system, which decreased the conduction-band minimum (CBM) of the first MOF and therefore its band gap. The decrease in the band gap considerably improved the photoconductivity of the MOF. The photocurrent response of the MOF and H_2_L is shown in Fig. 3. The largest photocurrent density of the MOF was approximately 8 × 10^−5^ mA cm^−2^, much larger than that of H_2_L (3 × 10^−5^ mA cm^−2^).

*Organic guest molecules* Besides the above researches where organic compounds directly serve as the ligands in photoconductive MOFs, donor–acceptor architecture can also be constructed completely with two different guest molecules with one as electron acceptor and the other as electron-donor. In this case, MOFs usually function as not only a host but also a photon antenna. Taking advantages of the highly ordered structure and permanent porosity of MOFs, a typical electron-accepting organic compound α,ω-dihexylsexithiophene (DH6T) and a typical electron-donating organic compound [6] -phenyl-C_61_-butyric acid methyl ester (PCBM) were infiltrated into the channel and cavity of MOF-177 (ZnO_4_(BTB)_2_; BTB = 1,3,5-benzenetribenzoate) [16]. The MOF in this MOF-donor–acceptor hybrid served as a host which confined and stabilized guest molecules, preventing their phase segregation, as well as a photon antenna which harvested light and transferred it to the guest acceptor molecules. This MOF-donor–acceptor hybrid provides another promising strategy that photoconductivity can be realized by carefully and appropriately designing the guest@MOF system.

#### Photoconductivity Based on Inorganic Moieties

**Fig. 4:**
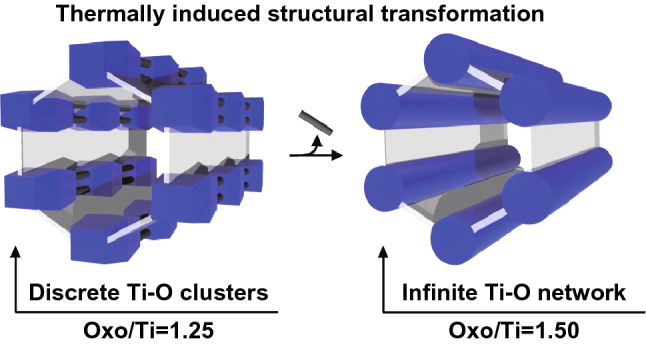
Thermally induced phase transformation of MIL-177. Reprinted with permission from Ref. [17]. Copyright 2018, Springer Nature

Although most reported photoconductive MOFs are based on the photoactive organic ligands, photoconductivity originated from inorganic building unit of MOFs has also been demonstrated. A mdip-based Ti-MOF with the formula Ti_12_O_15_(mdip)_3_(formate)_6_ (mdip = 3,3′,5,5′-tetracarboxydiphenylmethane), namely MIL-177-LT (LT stands for low temperature and HT below for high temperature), underwent an irreversible phase transformation into MIL-177-HT upon heating at 280 °C for 12 h, as shown in Fig. 4 [17]. The dimensionality change of the inorganic secondary building units in MIL-177 (LT: 0D; HT: 1D) had a significant impact on the photophysical properties. In contrast to MIL-177-LT which generated extremely weak photoconductivity signals upon ultraviolet (UV) laser irradiation due to the lack of conduction pathways in their frameworks, MIL-177-HT exhibited exceptional photoconductive response with the carrier mobility calculated to be at least 4 × 10^−4^ cm^2^ s^−1^ V^−1^, comparable to nano-sized TiO_2_ materials [18]. After phase transformation, MIL-177-HT exhibited a narrow band gap of 3.67 eV. This revealed that the band gap of MOFs could be lowered and photoconductivity could be increased by increasing the dimensionality of the inorganic building unit. MIL-177-HT was the first reported photoconductive MOFs whose photoconductivity mainly came from the inorganic Ti–O building unit. Further research on the conduction mechanism and the possible functions of inorganic building unit for photoconductivity is still under way.

### Applications

#### Solar Cells

As a kind of clean and reproducible energy, solar energy is expected to alleviate the energy crisis and reduce environment pollution induced by conventional fuels. Solar cells with high light harvesting and conversion efficiency are thus desired to optimize the energy structure. By converting light energy into electrical energy, photoconductive MOFs are promising for the construction of photoanodes in solar cells with higher efficiency, stability, and lower cost. Typically, a photoanode consists of a thin film of photosensitizer coated on porous metal oxide supported by a conductive and transparent substrate. The substrate widely used in photoanode is FTO glass. TiO_2_ and ZnO are the most commonly adopted metal oxides. And MOFs and derivatives have attracted much attention as photosensitizer in photoanodes.

It should be noted that while many MOFs have been reported to serve as functional additives or interlayers to modify the electrodes or electrolytes and improve charge generation and electrical conductivity in dye-sensitized solar cells, hybrid perovskite solar cells and organic solar cells, which has been summarized and discussed in an excellent review [19], investigations on photoconductive MOFs that directly serve as photoactive sensitizers in photoanodes are rare and limited. Most reported researches in this field focus on guest@MOFs systems, where the guest molecules like QDs, POM and dyes serve as photosensitizer to absorb photons and generate electrons and the MOF hosts better improve adsorption property and suppress charge recombination, which will be illustrated in detail as the following.

**Fig. 5:**
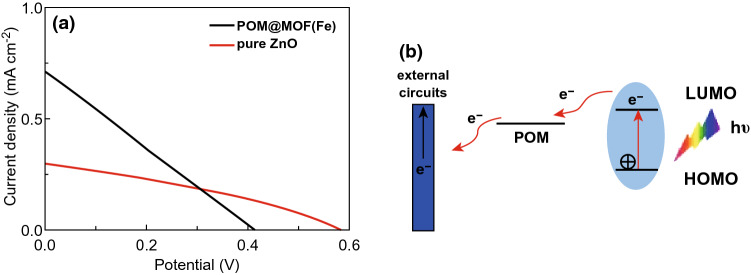
**a** *J*–*V* curve of cell with pure ZnO and POM@MOF(Fe)-modified ZnO photoanode. **b** Mechanism of charge transfer of POM@MOF(Fe). Reprinted with permission from Ref. [21]. Copyright 2017, Springer Nature

QDs are prominent photoactive materials with broad adsorption band and effective exciton generation and present a bright prospect for solar cells with relatively lower cost compared to silicon. A research innovatively combined CdTe QDs with MOF NTU-9, whose band gap is comparable to that of semiconductive TiO_2_ [20]. The CdTe/NTU-9 composite was used as photosensitizer in photoanode of a dye-sensitized solar cell and yielded a photoelectric conversion efficiency (PCE) up to 3.20%, much higher than 1.67% obtained with CdTe alone. The improved PCE was mainly ascribed to the enhanced adsorption capacity and lower charge recombination rate due to the ordered porous structure of NTU-9. Furthermore, polyoxometalate (POM), a kind of metal-oxide cluster compound, is an excellent electron acceptor with light-absorbing properties. POM@MOF hybrid is another effective system as photosensitizer for modification of photoanodes in solar cells. POM@MOF(Fe) hybrids were synthesized solvothermally by Zhang et al. and coated on ZnO photoanode [21]. As shown in Fig. 5a, compared to bare ZnO photoanode, the POM@MOF(Fe)-modified ZnO photoanode exhibited an increase in photoelectric conversion efficiency from 0.057 to 0.073%. Figure 5b illustrates the mechanism of charge transfer of POM@MOF(Fe). Upon irradiation, electrons were excited and transferred from the ligand of POM to the MOF(Fe) and then injected into the conduction band of ZnO. This process could enhance electron injection and electron–hole separation as well as photon capture, leading to higher photoelectric conversion efficiency.

Despite most researches on guest@MOFs systems with guest molecules as the photoactive sensitizer, some investigations on photoactive MOFs as the direct photosensitizer have been conducted as well. A graphene-doped Eu-MOF was synthesized to construct graphene-MOF/TiO_2_/FTO photoanode [22]. In this case, the photoactive Eu-MOF served as photosensitizer that adsorbed photons and generated electrons, and graphene facilitated charge transfer. Upon irradiation, electrons were generated from the LUMO level of the Eu-MOF and transferred through graphene to the conduction band of TiO_2_. The synergy of Eu-MOF and graphene accounted for the excellent photoconductivity of this fabricated photoanode, which presented a photoelectric conversion efficiency of 2.2%. Solar cells based on Pd-porphyrin Zn-SURMOFs 2 thin films grown through lay-by-layer method were fabricated and exhibited an efficiency of 0.45% [23]. As discussed above, porphyrin ligands are effective photoactive donors and in this solar cell, electrons were excited and injected from porphyrin ligand to the FTO substrate. An indirect band gap was observed in Pd-porphyrin Zn-SURMOFs 2, which strongly suppressed the electron–hole recombination, improving the photovoltaic device performance. However, in general, the photoelectric conversion efficiencies of photoanodes made from photoconductive MOFs in available researches are still relatively low. Further researches in future to obtain higher efficiency are needed.

#### Water Splitting

Water splitting consists of two half-cell reactions, namely hydrogen evolution reaction (HER) at cathode and oxygen evolution reaction (OER) at anode. To date, it is the major way to produce H_2_, which is a kind of renewable and eco-friendly energy helpful for environment protection and sustainable development. Conventionally, water splitting is realized through noble metal-based electrochemical catalysts like Pt, Ru, Ir and their oxides. However, the rareness in nature and high cost of noble metals limit their wide commercial applications. Photoelectrochemical (PEC) water splitting, which converts solar energy into chemical energy through photoactive electrodes, has attracted much attention due to a wide range of sources for the electrodes. Over the last few years, many MOFs have been utilized as cocatalysts [24–26] or interlayers at the semiconductor/electrolyte interface [27, 28] to improve photogenerated charge transfer or promote efficient charge injection at the semiconductor/electrolyte interface. In addition to MOFs as cocatalysts or interlayers, photoconductive MOFs have also investigated as photoanodes in photoelectrochemical water splitting.

For better PEC performance, the photoanode used in water splitting should possess outstanding light adsorption capacity, efficient charge separation and transfer properties and high stabilities. In general, photoanodes for photoelectrochemical water splitting fabricated from photoconductive MOFs possess several advantages: (i) a wide range of adsorption band in the Vis/near-IR range, which the wavelengths of solar light are mainly located within; (ii) the interface between MOF layer and the semiconducting substrate allows for effective charge injection from the MOF into the substrate and suppresses charge recombination, enhancing charge transfer rate; (iii) the porosity and large surface area of MOFs provide lots of active sites for OH-^−^ coordination and the ordered structure can retain stable in a long time. For example, the visible-light-responsive ZIF-67 was utilized to synthesize ZnO@Au@ZIF-67, which exhibited relatively high photoconversion efficiency up to 0.80% compared to ZnO@Au [29]. This was mainly ascribed to the visible-light adsorption of ZIF-67 and enhanced electron–hole separation. Upon irradiation, electrons were transferred from ZIF-67 shell to ZnO@Au core. Recently, Natarajan et al. have synthesized a Co(II)-MOF with a suitable band gap of 2.2–2.4 eV [30]. Upon irradiation, holes were generated in the d-valence band of the transition metal Co and facilitated the coordination of OH^−^ to the surface of MOF-based photoanode, accelerating charge transfer and water splitting. Notably, the crystal structure of photoconductive MOFs can affect the final H_2_ evolution activity. Two MOF compounds with different crystal structures were synthesized from 4′-(2,4-disulfophenyl)-3,2′:6′,3″-terpyridine (H_2_DSPTP) organic ligand and CuSO_4_·5H_2_O [31]. Although the two compounds shared the same ligand and metal ion, they exhibited different PEC performances. The structure with more extensive π–π interactions than the other facilitated photogenerated hole transfer and thus inhibited electron–hole recombination, enabling higher photoconversion efficiency. Therefore, crystal structure of photoconductive MOFs should also be taken into consideration for efficient photoelectrochemical H_2_ evolution.

## Photoluminescence

### Types of Photoluminescent MOFs

Recent years have seen tremendous progress in researches on MOFs with photoluminescent properties. In fact, many other materials such as lanthanide metals and molecule dyes also have been found to display photoluminescence. However, low absorption coefficient [32], aggregation-caused quenching (ACQ) [33], poor stability and other unavoidable defects prevent these traditional photoluminescent materials from large-scale applications in practical field. To overcome these defects, the most adopted strategy is to combine the exceptional porosity, stability and tunability of MOFs with the photoluminescence of conventional materials. The reported photoluminescence obtained in MOFs can be concluded as three types: linker-based luminescence, metal-centered luminescence and guest-induced luminescence.

#### Liker-Based Luminescence

*Ligand*-*centered luminescence* In some MOFs containing photoactive ligands, the photoluminescence is attributed to the intraligand emission or ligand-to-ligand charge or energy transfer, namely ligand-centered luminescence. In general, ligands with aromatic moieties possess more possibilities to realize photoluminescence because the conjugated π-electrons abundant in aromatic rings are easily excited to induce *π *− π* transition or facilitate charge transfer, which can lead to luminescent emission. Till now, many organic ligands have been investigated and most of them exhibit photoluminescence as expected, shown in Table 1.

**Table 1:** Organic ligands that enable photoluminescence

Organic ligands	Abbreviation used in text	References
1,10-phenanthroline-5,6-dione	PHDI	[96]
2,5-dihydroxyl-1,4-terephthalic acid	DHTA	[96]
4, 40-bis(pyridyl)diphenyl ether	BPDPE	[39]
3-(3,5dicarboxylphenyl)-5-(4-carboxylphenyl)-1-H-1,2,4-triazole	H_3_DBPT	[97]
1,1′,1″-(1,3,5-triazine-2,4,6-triyl)tripiperidine-4-carboxylic acid	H_3_TTPCA	[34]
Bismuth-1,3,5-benzenetricarboxylic acid	H_3_BTC	[35, 98]
*N*,*N*′-di(4-pyridyl)thiazolo-[5,4-d]thiazole	DPTTZ	[36]
3-(3′,5′-dicarboxylphenoxy)phthalic acid	H_4_L	[47]
para-terphenyl-3,30,5,50-tetracarboxylic acid	H_4_TPTC	[99]

**Fig. 6:**
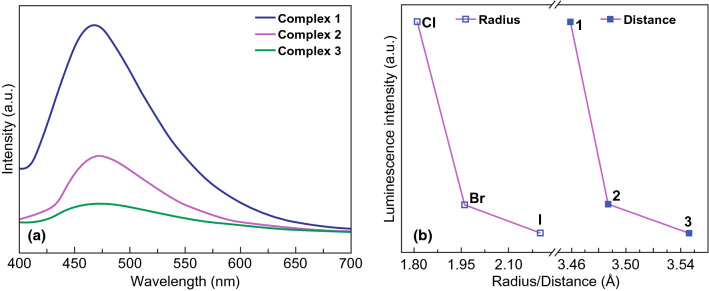
**a** Luminescence spectra of the three Pb-MOFs. **b** Relationship among luminescence intensity, halogen atom radius, and distance between ligands in the three Pb-MOFs. Reprinted with permission from Ref. [34]. Copyright 2019, American Chemical Society

It is proposed that the conformation of ligands mainly influences the emission band and that the luminescence intensity can be modulated by the distance between neighboring ligands. This was confirmed by the research on a series of H_3_TTPCA-based Pb-MOFs that had different compositions of metal oxygen clusters [Pb_7_(COO)_12_X_2_] (X = Cl, Br, or I) [34]. The emission spectrums of the three MOFs and free H_3_TTPCA molecule under 371 nm excitation were compared. As shown in Fig. 6a, the emission band shifted from 440 for H_3_TTPCA to 467 nm for Pb-MOFs, which was attributed to the increase in the conformation of the organic ligand from one in free H_3_TTPCA to three in the MOFs. The effect of conformation of organic ligand was also confirmed by the red shift presented by Bi-MOF [Bi(BTC)(H_2_O)]·H_2_O compared to H_3_BTC [35]. Red-shifted luminescence band caused by increased ligand conformation may be due to the reduced molecule vibration and decreased loss of energy by radiationless decay. Furthermore, Fig. 6b shows that with an increase in the halogen atom radius in the three Pb-MOFs, the distances of organic ligand in the three MOFs increased as well, which accounted for the decrease in the luminescence intensities at 467 nm of the three MOFs as observed in Fig. 6a. The photoluminescence of the three MOFs originated from π* − *π* transition of the organic ligand, and thus, an increase in ligand distance should negatively influence inter-ligand charge transfer and thereby result in lower luminescence intensity. Comparisons between the synthetic and activated (heated under vacuum at 100 °C for 4 h) MOFs further evidenced the relationship between ligand distance and luminescence intensity. After activated, two MOFs exhibited increased ligand distance and decreased luminescence intensity, while the other exhibited decreased ligand distance and enhanced luminescence intensity.

**Fig. 7:**
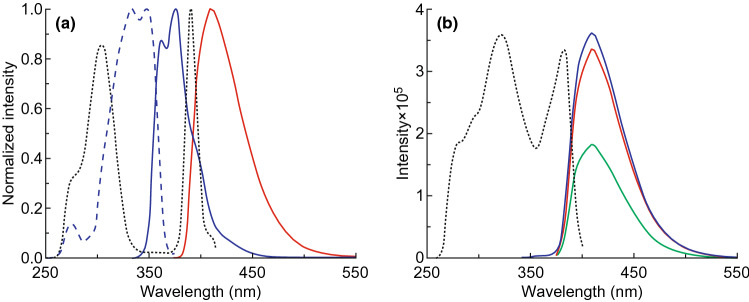
**a** Excitation (dashed lines) and emission (solid lines) spectra of free NDC (blue) and DPTTZ (red) ligands showing the requisite spectral overlap for FRET. **b** Excitation (dashed black line) and emission spectra of Zn_2_(NDC)_2_(DPTTZ) showing exclusively DPTTZ-centric emission regardless of excitation wavelengths (*λ*_Ex_ = 320 (blue), 350 (green), and 380 nm (red)). Reprinted with permission from Ref. [36]. Copyright 2019, American Chemical Society. (Color figure online)

Sometimes in order to enhance the luminescence intensity or fluorescence changes in MOFs for detection and sensing, another organic ligand is introduced as an antenna that absorbs more light and transfers the energy to the emissive ligand. For MOF Zn_2_(NDC)_2_(DPTTZ), naphthalene dicarboxylate (NDC) serves as an antenna and energy donor and *N*,*N*′-di(4-pyridyl)thiazolo-[5,4-d]thiazole (DPTTZ) functions as energy acceptor and light emitter [36]. As shown in Fig. 7a, a good overlap between the adsorption spectra of NDC and the emission spectra of DPTTZ was observed, which was the prerequisite for Förster resonance energy transfer from NDC to DPTTZ. Figure 7b shows the exclusively DPTTZ-centric emission in spite of excitation wavelengths, which was rarely observed in other photoluminescent MOFs. What is more, compared to free DPTTZ ligand, Zn_2_(NDC)_2_(DPTTZ) exhibits more efficient fluorescence changes in the presence of Hg^2+^ under illumination at a wide wavelength region, making it a possible sensor for Hg^2+^.

*Ligand*-*to*-*metal charge transfer* As one of the possible ways to realize photoluminescence in MOFs, ligand-to-metal charge transfer is due to the interactions between metal ions and organic ligands. It is typically observed in Pb/Zn/Cu-based MOFs, where the metal center binds to O atoms of the organic ligand and charge transfer occurs from ligand to metal through metal–oxygen bonds. For example, [Pb(H_2_O)(γ-CD)](NO_3_)_2_·11H_2_O (γ-CD–Pb), obtained from γ-cyclodextrin and Pb^2+^, exhibited photoluminescence with maximum emission wavelength at 345 nm excited at 290 nm [37]. While cyclodextrins are non-aromatic ligands and lack photoluminescence, it was the presence of Pb(II) that induced charge transfer from ligand to metal center through Pb–O bonds under irradiation and hence photoluminescence. In Zn_3_·BDC·2BTC·2NH(CH_3_)_2_·2NH_2_(CH_3_)_2_, a new emission peak at 430 nm was attributed to charge transfer from O atoms of the ligands to the empty 4 s orbitals of Zn^2+^ [38]. The aforementioned photoconductive ZnL(DPE)(H_2_O)·H_2_O [15] also exhibited a weak photoluminescence band at 450 nm due to ligand-to-metal charge transfer in the presence of N-donor ligand DPE. However, the luminescence intensity of this MOF was much weaker than the free H_2_L ligand, since the MOF exhibited reduced charge recombination which improved the photoconductivity but inhibited photoluminescence. Interestingly, ligand-to-metal charge transfer can also occur between the ligand of MOFs and metal ions in the environmental solutions. A new emission band was observed in [CuI(BPDPE)]_n_ when treated with Al^3+^ solutions, making it a possible Al^3+^ sensor [39], which will be discussed in detail in the following.

**Fig. 8:**
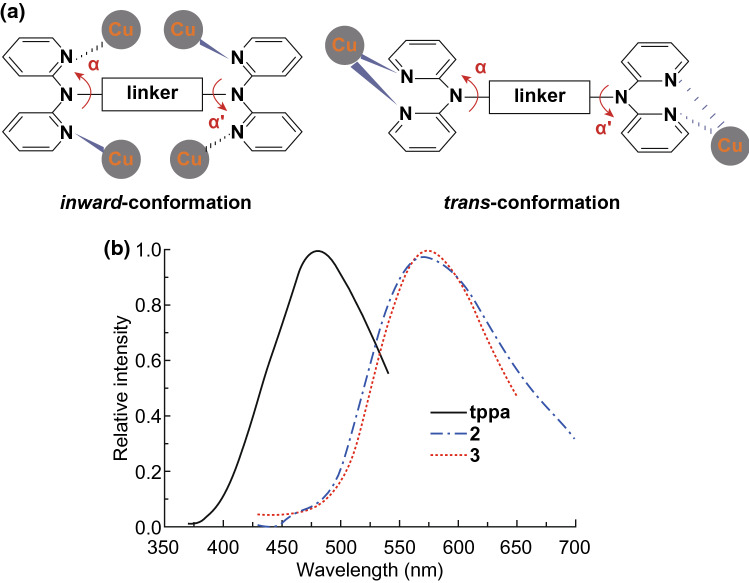
**a** *Inward*- and *trans*-conformations of [Cu_6_(tppa)(μ_3_-Br)_6_]_n_ and [Cu_2_(tppa)(μ-CN)_2_]_n_,. **b** Emission spectra of free tppa ligand, [Cu_6_(tppa)(μ_3_-Br)_6_]_n_ (2) and [Cu_2_(tppa)(μ-CN)_2_]_n_ (3). Reprinted with permission from Ref. [40]. Copyright 2012, Royal Society of Chemistry

*Metal*-*to*-*ligand charge transfer* Photoluminescence in some MOFs originates from metal-to-ligand charge transfer. Typically, metal-to-ligand charge transfer involves *π*-rich ligands which serve as effective electron acceptors in MOFs. The metal involved in metal-to-ligand charge transfer is mainly d^10^ Cu(I), whose d electrons are right in the valence orbitals to facilitate charge transfer. The emissions of a series of Cu(I) MOFs of 2,2′-dipyridylamine derivatives, formulated as [Cu_6_(tppa)(μ_3_-Br)_6_]_n_, [Cu_2_(tppa)(μ-CN)_2_]_n_, [Cu(tpbpa)Br]_n_, [Cu_4_(tpbpa)_2_(μ-I)_4_]_n_, [Cu_4_(tpbpa)(μ-CN)_4_]_n_ and [Cu_8_(tpbpa)(μ-CN)_8_]_n_·2nH_2_O, were all ascribed to metal-to-ligand charge transfer due to the presence of π-rich ligands with a lower energy of the π*-orbital, which were more prone to induce metal-to-ligand charge transfer [40]. Compared to the corresponding free ligands, the emission bands of these MOFs were all red-shifted but in various degrees due to different ligand conformations, indicative of the influence of ligand conformations on the emission bands. For example, the tppa ligands in [Cu_6_(tppa)(μ_3_-Br)_6_]_n_ and [Cu_2_(tppa)(μ-CN)_2_]_n_ adopted inward- and trans-conformations, respectively, as shown in Fig. 8a. The emission bands of these two MOFs shown in Fig. 8b revealed different emission peaks at 569 and 573 nm, respectively.

#### Metal-Centered Luminescence

Lanthanide is well known to be a series of metals that exhibit exceptional photoluminescent properties such as large Stoke shift, extremely sharp emission and long life time, owing to the unique *f*–*f* transitions between the 4f electrons. However, the low absorption coefficient of lanthanide metals hinders their wide applications in practical luminescent devices. One feasible strategy to overcome this defect is to combine lanthanide metals with MOFs, which afford effective energy donor organic ligands that serve as an antenna with excellent light absorption properties. Besides, some rare earth metals also exhibit photoluminescence under irradiation. Therefore, many metal-centered luminescent MOFs have been successfully synthesized by directly constructing frameworks with lanthanide or rare earth metals or by doping non-luminescent MOFs with lanthanide or rare earth metals, as shown in Table 2.

**Table 2:** Metals involved in MOFs to induce photoluminescence

Metal centers	Characteristic emissions	Synthesized MOFs	References
Tb	^5^D_4_ → ^7^F_6_ 489 nm ^5^D_4_ → ^7^F_5_ 543 nm ^5^D_4_ → ^7^F_4_ 582 nm ^5^D_4_ → ^7^F_3_ 623 nm	MR-MOF-Tb WR-MOF-Tb	[42]
		Tb-SA	[32]
		{[Tb(L)(H_2_O)_2_]·5H_2_O}_n_	[41]
		[Tb(TCBA)(H_2_O)_2_]_2_·DMF	[100]
		{[Me_2_NH_2_^+^][Tb(L)(H_2_O)_2_]}_n_	[101]
		[TbL_2_(H_2_O)_4_]_n_·nNO_3_	[102]
		TbTMA	[103]
Eu	^5^D_0_ → ^7^F_0_ 579 nm ^5^D_0_ → ^7^F_1_ 590 nm ^5^D_0_ → ^7^F_2_ 614 nm ^5^D_0_ → ^7^F_3_ 650 nm ^5^D_0_ → ^7^F_4_ 697 nm	MR-MOF-Eu WR-MOF-Eu	[42]
		Eu(Ln)@bio-MOF-1	[52]
		{[Eu(L)(H_2_O)_2_]·5H_2_O}_n_	[41]
		[Eu_2_(SO_4_)_2_(H_6_htp)(H_2_O)_4_]·10H_2_O	[104]
		{[Eu(2,5-FDA)_0.5_(Glu)(H_2_O)_2_]·xH_2_O}_n_	[105]
		Zn(BDC)(dpNDI): 2% Eu	[106]
		{(Me_2_NH_2_^+^)[Eu(L)(H_2_O)_2_]}_n_	[101]
		[EuL_2_(H_2_O)_4_]_n_·nNO_3_	[102]
Sm	^4^G_5/2_ → ^6^F_5/2_ 561 nm ^4^G_5/2_ → ^6^F_7/2_ 596 nm ^4^G_5/2_ → ^6^F_9/2_ 644 nm ^4^G_5/2_ → ^6^F_11/2_ 703 nm	MR-MOF-Sm WR-MOF-Sm	[42]
		[Sm_2_(SO_4_)_2_(H_6_htp)(H_2_O)_4_]·10H_2_O	[104]
		{[Sm(2,5-FDA)_0.5_(Glu)(H_2_O)_2_]·xH_2_O}_n_	[105]
		{(Me_2_NH_2_^+^)[Sm(L)(H_2_O)_2_]}_n_	[101]
		[SmL_2_(H_2_O)_4_]_n_·nNO_3_	[102]

**Fig. 9:**
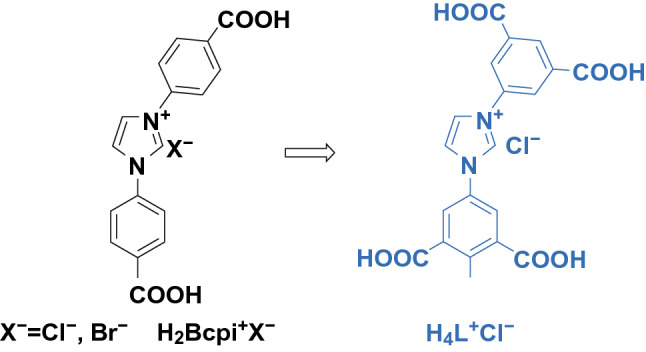
Modification of H_2_Bcpi^+^X^–^ for H_4_L^+^Cl^–^. Reprinted with permission from Ref. [41]. Copyright 2019, American Chemical Society

It should be noted that it is crucial to choose appropriate organic ligands for synthesis of metal-centered photoluminescent MOFs. Aromatic ligands with a *π*-conjugated system or a heterocyclic organic ligand are ideal ligands to this end, and to better improve their light adsorption capacity, carboxylic groups are extensively utilized to modify the ligands. In the synthesized MOFs, organic ligands function as antennas and sensitizers, which effectively adsorb light and transfer the energy to the metal center. H_4_L^+^Cl^−^ ligand, for example, was prepared by modifying H_2_Bcpi^+^X^−^ ligand with imidazole and two aromatic carboxylic acids, as shown in Fig. 9 [41]. The as-synthesized H_4_L^+^Cl^−^ ligand exceptionally met the demand for antennas in photoluminescent MOFs. A series of LnMOFs formulated as {[Ln(L)(H_2_O)_2_]·5H_2_O}_n_ (Ln = Eu, Tb, Gd, and Eu_*x*_Tb_1−*x*_) with superior photoluminescent properties were thus synthesized based on H_4_L^+^Cl^−^ ligand.

**Fig. 10:**
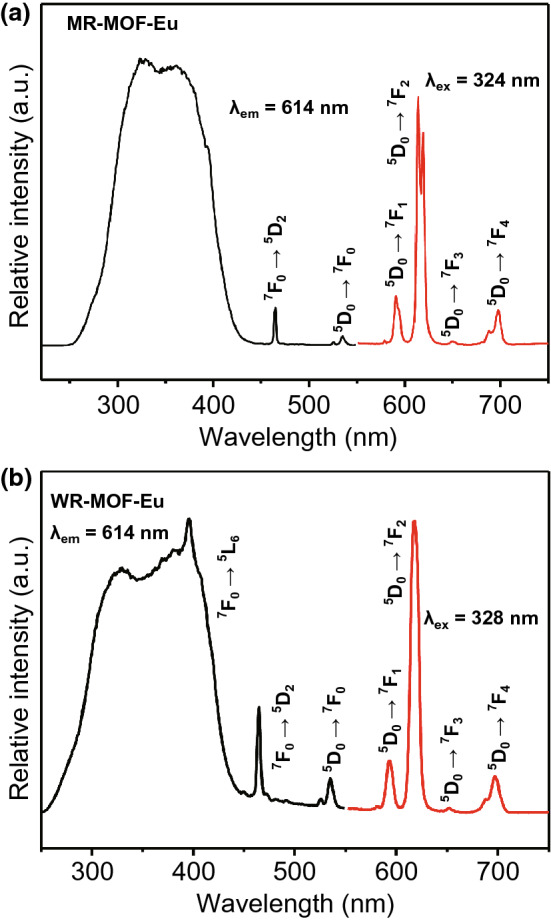
Excitation (black line) and emission (red line) spectra of **a** MR-MOF-Eu and **b** WR-MOF-Eu hybrid materials. Reprinted with permission from Ref. [42]. Copyright 2020, Elsevier. (Color figure online)

**Fig. 11:**
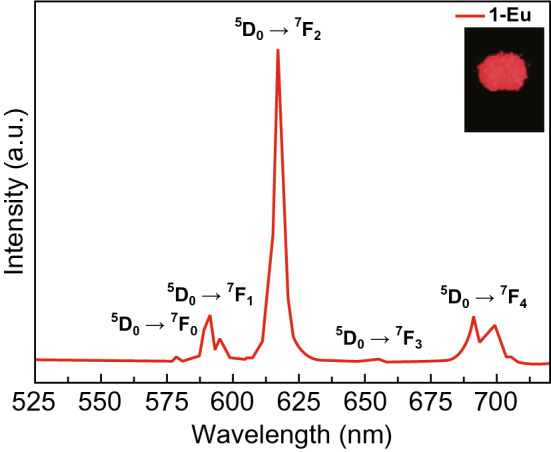
Solid-state emission spectrum of {[Eu(L)(H_2_O)_2_]·5H_2_O}_n_ at 254 nm at room temperature. Reprinted with permission from Ref. [41]. Copyright 2019, American Chemical Society

In general, metal-centered luminescent MOFs inherit the luminescent properties of the original metals, as both of them have the similar emission bands. Most of LnMOFs can emit light with several colors, corresponding to a number of different transitions from excitation state to ground state characteristic of lanthanide metals. For example, two hybrids of MR-MOF-Eu and WR-MOF-Eu synthesized through assembly of MOFs based on Eu metal ions and 2-amino-1,4-benzendicarboxylic acid (NH_2_-BDC) with two microsphere resins (Wang resin (WR) and Merrifield resin (MR)) exhibited similar emission bands at around 579, 590, 614, 650, and 697 nm due to characteristic ^5^D_0_ → ^7^F_J_ (J = 0–4) transitions of Eu^3+^, as shown in Fig. 10a, b [42]. {[Eu(L)(H_2_O)_2_]·5H_2_O}_n_ based on the aforementioned H_4_L^+^Cl^−^ ligand [41] also presented similar emission bands at 579, 581, 617, 653, and 697 nm, as shown in Fig. 11. Photoluminescent MOFs with the same lanthanide metal possess similar emission bands due to their characteristic state transitions. Notably, different lanthanide metals can be combined into one MOF to obtain a combination of different luminous colors. Modulation of the proportions can result in tunable colors and even white light emission, which will be discussed in detail in the following. The characteristic emission bands of commonly used lanthanide metals are listed in Table 3 along with recently synthesized MOFs.

**Table 3:** Photoluminescent sensors based on MOFs toward various analytes

Target analyte	Luminescent MOFs as sensors	*K*_SV_ (M^−1^)	Limit of detection (LOD)	References
Fe^3+^	Bio-MOF-1@RhB	5.5 × 10^4^	1.1 ppm	[33]
	[Pb_1.5_(DBPT)]_2_·(DMA)_3_(H_2_O)_4_	1.2 × 10^5^	2.5 ppm	[97]
	[Bi(BTC)(H_2_O)]·H_2_O	2.02 × 10^4^	1.59 μM	[35]
	{[Cd_1.5_(DBPT)(DiPyDz)(H_2_O)]·3.5H_2_O}_n_	4.789 × 10^5^	78 ppb	[58]
	[Cd(L)(pda)]	1.03 × 10^5^	112 ppb	[59]
	{[Tb(Cmdcp)(H_2_O)_3_]_2_(NO_3_)_2_·5H_2_O}_n_	5.532 × 10^3^	4.0 μM	[56]
	[Zn(1,6-NDS)(bbimb)_1.5_]·2H_2_O	7.17 × 10^3^	1.76 × 10^−4^ M	[107]
	[Cd_2_(1,6-NDS)_2_(bbimb)_3_(H_2_O)_4_]·2H_2_O	1.01 × 10^4^	1.80 × 10^−4^ M	[107]
Cu^2+^	Tb-SA	6.298 × 10^3^	1 × 10^−4^ M	[32]
	PEG-ZnS QDs@ZIF-67	–	0.96 nM	[45]
Al^3+^	[CuI(BPDPE)]_n_	1.2560 × 10^4^	2.1 × 10^−6^ M	[39]
	[Pb_1.5_(DBPT)]_2_·(DMA)_3_(H_2_O)_4_	4.3 × 10^4^	–	[97]
Cr_2_O_7_^2−^	[Bi(BTC)(H_2_O)]·H_2_O	1.95 × 10^4^	1.64 μM	[35]
	[Cd(L)(pda)]	1.01 × 10^5^	126 ppb	[59]
Zr^4+^	[Pb_1.5_(DBPT)]_2_·(DMA)_3_(H_2_O)_4_	1.6 × 10^5^	–	[97]
In^3+^	[Pb_1.5_(DBPT)]_2_·(DMA)_3_(H_2_O)_4_	1.6 × 10^5^	–	[97]
O_2_	MIL-100(In) ⊃ Tb^3+^	7.59	0.4%	[108]
TBBPA (tetrabromo-bisphenol A)	MOF-74(Zn)-en	–	0.75 μg L^−1^	[53]
nitrobenzene	Zn_3_(BTC)_2_: 4%Eu(III)	3.957 × 10^3^	0.97 ppm	[51]
NP(p-nitrophenol)	In-atp	–	2 × 10^−3^ U L^−1^	[48]
picric acid(TNP)	[CuI(BPDPE)]_n_	1.5 × 10^4^	1.09 × 10^−6^ M	[39]
2,4-dinitrophenol	[Zn(H_2_L)(2,2-bipy)]_n_	1.83 × 10^4^	7.08 × 10^−4^ mM	[47]
acetone	{[Cd_1.5_(DBPT)(DiPyDz)(H_2_O)]·3.5H_2_O}_n_	–	0.0013% (v/v %)	[58]
DMA (*N*,*N*-dimethylacetamide)	[Pb_1.5_(DBPT)]_2_·(DMA)_3_(H_2_O)_4_	–	–	[97]
triiodothyronine hormone (T3)	Cu-MOF-NPs	–	0.198 ng dL^−1^	[50]
l-cysteine	{[Ca_1.5_(μ_8_-HL_1_)(DMF)_2_]·DMF}_n_	–	15 nM	[109]
Alpha-fetoprotein (AFP)	Cu-MOF-NPs	–	1.18 ng mL^−1^	[49]

#### Guest-Induced Photoluminescence

Many molecules are well photoluminescent but with poor stability and intrinsic aggregation-caused quenching effect, hindering their practical applications. Encapsulation of these molecules into the pores of MOFs, where the guest molecules are isolated from each other to avoid aggregation-caused quenching effect, has been adopted to obtain photoluminescent MOFs. The rigid structure of MOFs also provides protection for luminescent molecules and enhances the material stability. A large class of these guest molecules is organic dyes such as cyanine and rhodamines [33]. Others include perovskites like MAPbBr_3_ [43], quantum dots [44], and so forth.

**Fig. 12:**
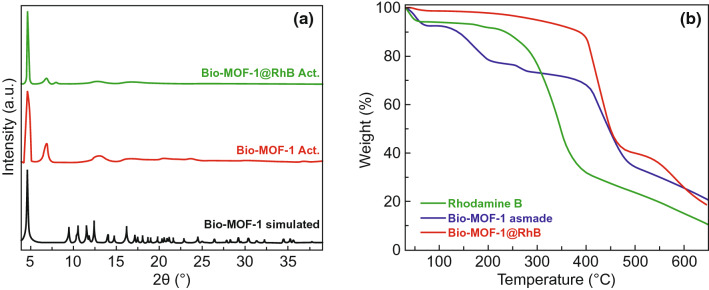
**a** PXRD pattern of Bio-MOF-1 simulated (black), Bio-MOF activated (brown) & Bio-MOF@RhB activated (green). **b** TGA profiles of as-made Bio-MOF-1 (blue), RhB (green) & Bio-MOF@RhB (brown). Reprinted with permission from Ref. [33]. Copyright 2020, Elsevier. (Color figure online)

A recent research conducted by Let et al. adopted rhodamine B (RhB) and Bio-MOF-1 to synthesize dye@MOF composite [33]. RhB was chosen because of its cationic nature, exceptional photoluminescence and abundant free carboxylic groups to interact with Fe^3+^. The anionic nature of Bio-MOF-1 framework facilitated effective bonds with cationic RhB molecules in an ion-exchange process. As shown in Fig. 12a, the PXRD profiles of as-synthesized Bio-MOF-1 and Bio-MOF-1@RhB exhibited minimal difference, indicating that the pristine MOF structure was nearly unaffected upon the encapsulation of RhB molecules. Figure 12b displays the TGA profiles of RhB, Bio-MOF-1 and Bio-MOF-1@RhB. While RhB molecules experienced substantial loss after ~ 300 °C, Bio-MOF-1@RhB showed no obvious loss up to 400 °C, indicating that the confinement effect of MOF could significantly enhance the stability of RhB, enabling its application in Fe^3+^ detection.

Quantum dots (QDs) are small particles with superior photoactive properties. PEG-ZnS QDs@ZIF-67 nanohybrids were synthesized through encapsulation of polyethylene glycol (PEG)-capped ZnS quantum dots into ZIF-67 [45]. The adsorption properties of ZIF-67 to capture and concentrate Cu^2+^ facilitated ZnS QDs to detect Cu^2+^ as selective luminescent sensor. Notably, compared to traditional QDs like CdSe and PbSe, carbon quantum dots (C-QDs) possess more attractive advantages such as higher stability, lower toxicity and so forth. A C‑QDs@UiO-66-(COOH)_2_ composite film was fabricated through electrophoretic deposition and served as luminescent temperature sensor [46]. In addition to QDs, photoactive perovskites can not only improve photoconductivity in solar cells, but also promote photoluminescence in MOFs. However, the inherent instability inhibits their practical applications. For example, organic MA^+^ cations of MAPbX_3_ (MA = CH_3_NH_3_, X = Cl, Br, I) undergo rapid degradation in polar solutions or high temperature. Encapsulating MAPbX_3_ into MOFs can greatly enhance its stability, and its application in information protection has been demonstrated [43]. QDs and perovskites as guest molecules to promote photoluminescent properties of MOFs for light emitting and optical information protection will be further discussed in the following part.

### Applications

#### Photoluminescent Sensors

**Fig. 13:**
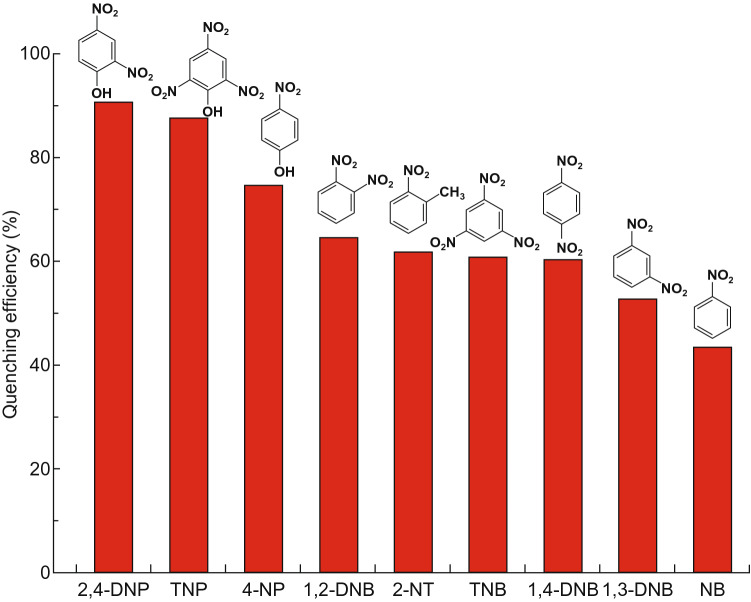
The quenching percentage of [Zn(H_2_L)(2,2-bipy)]_n_ in DMF of NB, 2-NP, 4-NP, 1,2-DNB, 1,3-DNB, 1,4-DNB, 2,4-DNP, TNP and TNB at excitation of 278 nm. Reprinted with permission from Ref. [47]. Copyright 2019, Royal Society of Chemistry

Photoluminescent MOFs as sensors are expected to play an important role in many fields such as industrial production, environmental protection and health care. Many photoluminescent MOFs exhibit high sensitivity toward specific metal ions or substances harmful to environment or even human body. Photoluminescent MOFs as sensors reported recently are shown in Table 3. In particular, nitro explosives (NEs) have been widely used in industrial production, but they can cause lots of problems, not only environmental pollution but also threat to human health and even country security. Effective NEs detections are in great demand, and photoluminescent MOFs have attracted much attention in this field. For example, [Zn(H_2_L)(2,2-bipy)]_n_ based on H_4_L ligand exhibited ligand-centered photoluminescence and was highly promising for detections of a series of nitroaromatic explosives, among which the detection of 2,4-dinitrophenol (2,4-DNP) could reach a high *K*_sv_ value of 1.83 × 10^4^ M^−1^ and a low detection limit of 7.08 × 10^−4^ mM, as shown in Fig. 13 [47]. In addition, luminescent MOFs can also function as biosensors in biological field. For instance, the alkaline phosphatase (ALP) enzyme, as a signal for serious diseases, can be detected in human serum samples by In-atp [48]. Similarly, Cu-MOF-NPs can detect alpha-fetoprotein (AFP) for liver cancer diagnosis [49] as well as triiodothyronine hormone (T3) for thyroid disease diagnosis [50]. More recently reported luminescent MOF sensors toward various metal ions and organic molecules are listed in Table 3, and the mechanisms for luminescent sensing are discussed as following.

**Fig. 14:**
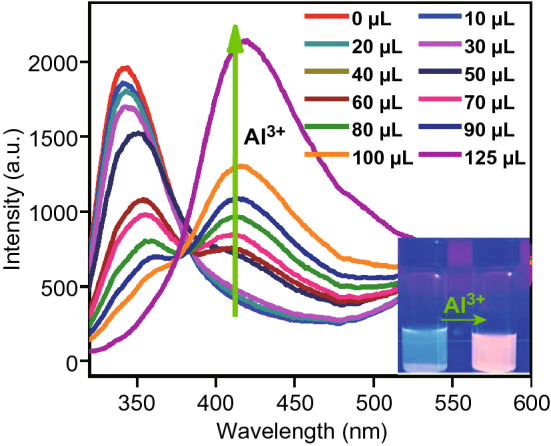
Changes of emission intensities of [CuI(BPDPE)]_n_ with incremental addition of Al^3+^ (1 mM). Reproduced with permission from Ref. [39]. Copyright 2020, Elsevier

It has been widely observed that exposure to specific analytes can arise changes in the intensity of luminescence emitted by MOFs. The changes may be luminescence quenching (turned off) or enhancement (turned on) or sometimes even both, which can be explained by the theory of charge and energy transfer. One typical example is the aforementioned Cu(I)-MOF [CuI(BPDPE)]_n_, which presented an emission peak at 340 nm under excitation at 305 nm [39]. Treated with Al^3+^ of increasing concentration, the emission intensity at 340 nm gradually decreased and the intensity of a new emission peak at 420 nm gradually got stronger, as shown in Fig. 14. This phenomenon could be ascribed to the change of charge transfer from ligand-to-ligand (BPDPE-to-BPDPE) to ligand-to-metal (BPDPE-to-Al^3+^) as a result of Al–O weak interactions in the presence of Al^3+^.

Many photoluminescent MOFs display luminescent quenching effect when exposed to external ions or molecules. The quenching effect can be analyzed by the Stern–Völmer equation:

1 $$ I_{0} /I \, = \, 1 \, + \, K_{\text{SV}} \left[ M \right] $$

where *I*_0_ refers to the pristine luminescence intensity of MOFs, *I* is the luminescence intensity of MOFs after being treated with external analytes, [*M*] denotes the concentration of the external analytes, and *K*_SV_ is the quenching constant of MOFs. In terms of the quenching process, luminescent quenching can be divided into two parts: dynamic quenching and static quenching. While for some luminescence quenching only one type of process either dynamic or static is involved, most quenching processes include both, such as the coexistence of dynamic and static quenching when an Eu(III)-doped Zn-MOF, Zn_3_(BTC)_2_: 4%Eu(III), was exposed to nitrobenzene (NB) [51].

**Fig. 15:**
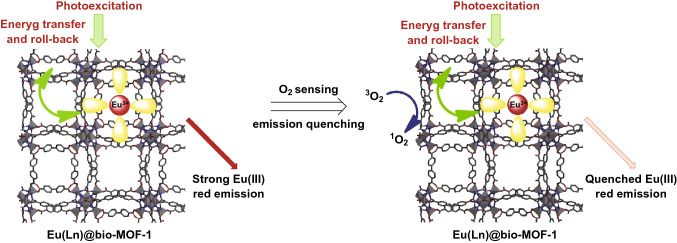
Sensing mechanism of Eu(Ln)@bio-MOF-1 composite samples toward O_2_. Reproduced with permission from Ref. [52]. Copyright 2019, Elsevier

Dynamic quenching originates from the interactions between the energy donor and quencher. For example, the sensing mechanism of Eu(Ln)@bio-MOF-1 toward O_2_ was confirmed as the O_2_ quenching on long-range energy roll-back from ligand triplet state to bio-MOF-1, as depicted in Fig. 15 [52]. Upon irradiation, the bio-MOF-1 matrix absorbed photons and transferred the energy to the organic diamine ligands of Eu(III) complexes, which further transferred the energy to emissive Eu(III) ions, resulting in strong red emission. There was supposed to be an energy roll-back procedure from ligand to bio-MOF-1. Due to the fully matched multiplicity, ^3^O_2_ could quench the energy roll-back procedure, accompanied by the release of ^1^O_2_, leading to the quenching effect of luminescence of Eu(Ln)@bio-MOF-1.

In contrast, static quenching results from the generation of non-luminescent complexes between the fluorophore and quencher, diminishing the energy transfer between the fluorophore and energy donor. A LnMOF thin film of terbium-succinate (Tb-SA) was fabricated through cathodic electrodeposition and showed highly eye-detectable luminescent response as a photoluminescent sensor for Cu^2+^ in aqueous environment as well as high sensitivity, selectivity and stability [32]. The bright green light at 545 nm emitted from the film excited by laser at 303 nm could be quenched as a result of static quenching induced by non-luminescent complex of Cu^2+^ and succinic acid due to possible ion-exchange between Cu^2+^ and Tb^3+^, which inhibited the energy transfer in Tb-SA composite.

**Fig. 16:**
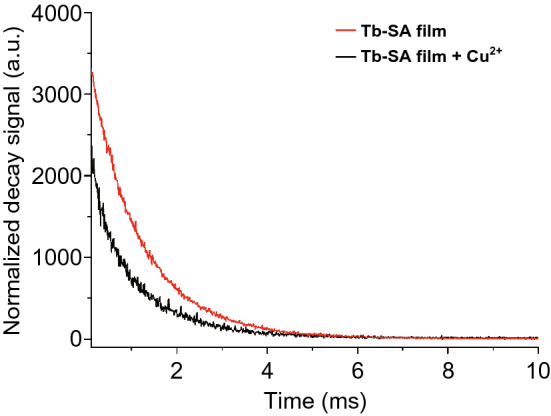
Luminescence decay curves of Tb-SA films with and without the addition of Cu^2+^. Reproduced with permission from Ref. [32]. Copyright 2015, Elsevier

**Fig. 17:**
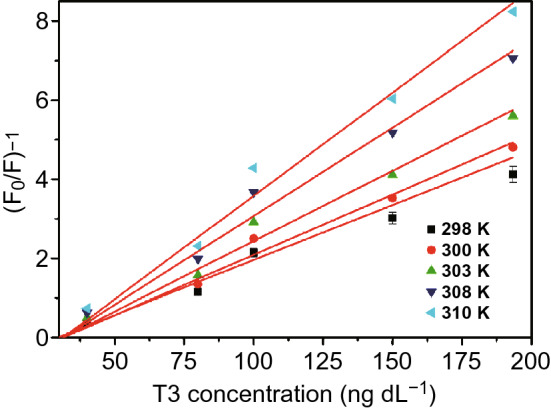
The Stern–Völmer plots for PL quenching of Cu‐MOF‐NPs by T3 hormone at different five temperatures. Reproduced with permission from Ref. [50]. Copyright 2019, John Wiley & Sons

There are two ways to distinguish static and dynamic quenching: through the luminescence lifetime or through *K*_SV_ response toward temperature change. On the one hand, luminescence lifetime retains nearly unchanged after static quenching, while in the case of dynamic quenching, increase in concentration of quencher gives rise to decrease in luminescence lifetime. The aforementioned Tb-SA exhibited static quenching in the presence of Cu^2+^ since the luminescence lifetimes calculated with and without Cu^2+^ were nearly equal to each other, as shown in Fig. 16 [32]. The dynamic quenching of Eu(Ln)@bio-MOF-1 toward O_2_ was confirmed by the fact that the lifetime of Eu(III) emission obviously decreased when O_2_ concentrations increased from 0 to 100% [52]. On the other hand, in dynamic quenching the K_SV_ value increases with increased temperature, and for static quenching, the converse is true. For instance, the luminescence quenching induced by triiodothyronine hormone (T3) in Cu-MOF-NPs investigated by Sheta et al. was ascribed to dynamic quenching because the *K*_SV_ value was positively proportional to the temperature, as shown in Fig. 17, where the slopes of simulated lines stand for the *K*_SV_ values [50].

**Fig. 18:**
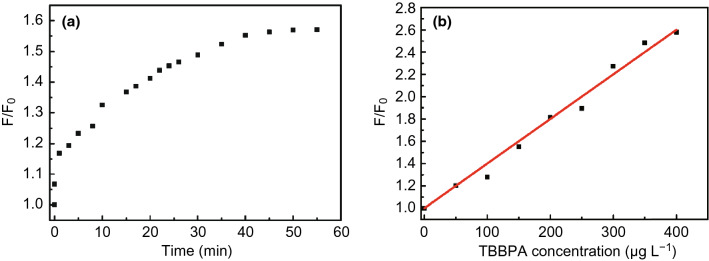
**a** Photoluminescence enhancement ratio of MOF-74(Zn)-en in the presence of TBBPA (150  μg L^−1^) at different incubation time. **b** Calibration curves of photoluminescence enhancement ratios of MOF-74(Zn)-en in the presence of different concentrations of TBBPA (0–400  μg L^−1^). Reproduced with permission from Ref. [53]. Copyright 2019, Elsevier

While many luminescent sensors are fabricated based on quenching effect, others are based on the phenomenon of luminescence enhancement or generation of a new emission band. MOF-74(Zn)-en could act as a highly selective sensor for TBBPA [53]. Increases of both the concentration of TBBPA and interaction time resulted in enhanced fluorescence intensity of MOF-74(Zn)-en. The results shown in Fig. 18 revealed that the optimal contact time for TBBPA detection was around 40 min and the simulated Stern–Völmer equation was *F*/*F*_0_ = 0.004[*C*_TBBPA_] + 1 (*R*^2^ = 0.998). The possible mechanism could be attributed to Förster resonance energy transfer from MOF-74(Zn)-en to TBBPA as TBBPA could interact with amino groups in MOF-74(Zn)-en and an overlap between the adsorption spectrum of MOF-74(Zn)-en and the emission spectrum of TBBPA was observed, enabling fluorescence enhancement. As for highly luminescent Zn_2_(bpdc)_2_(bpee) MOF (H_2_(bpdc) = 4,4′-biphenyldicarboxylic acid and bpee = 1,2-bipyridylethene), exposure to subppm amines turned on a new absorption band and a new luminescence band due to the release of bpee molecules exchanged by amines, enabling sensing functions [54].

**Fig. 19:**
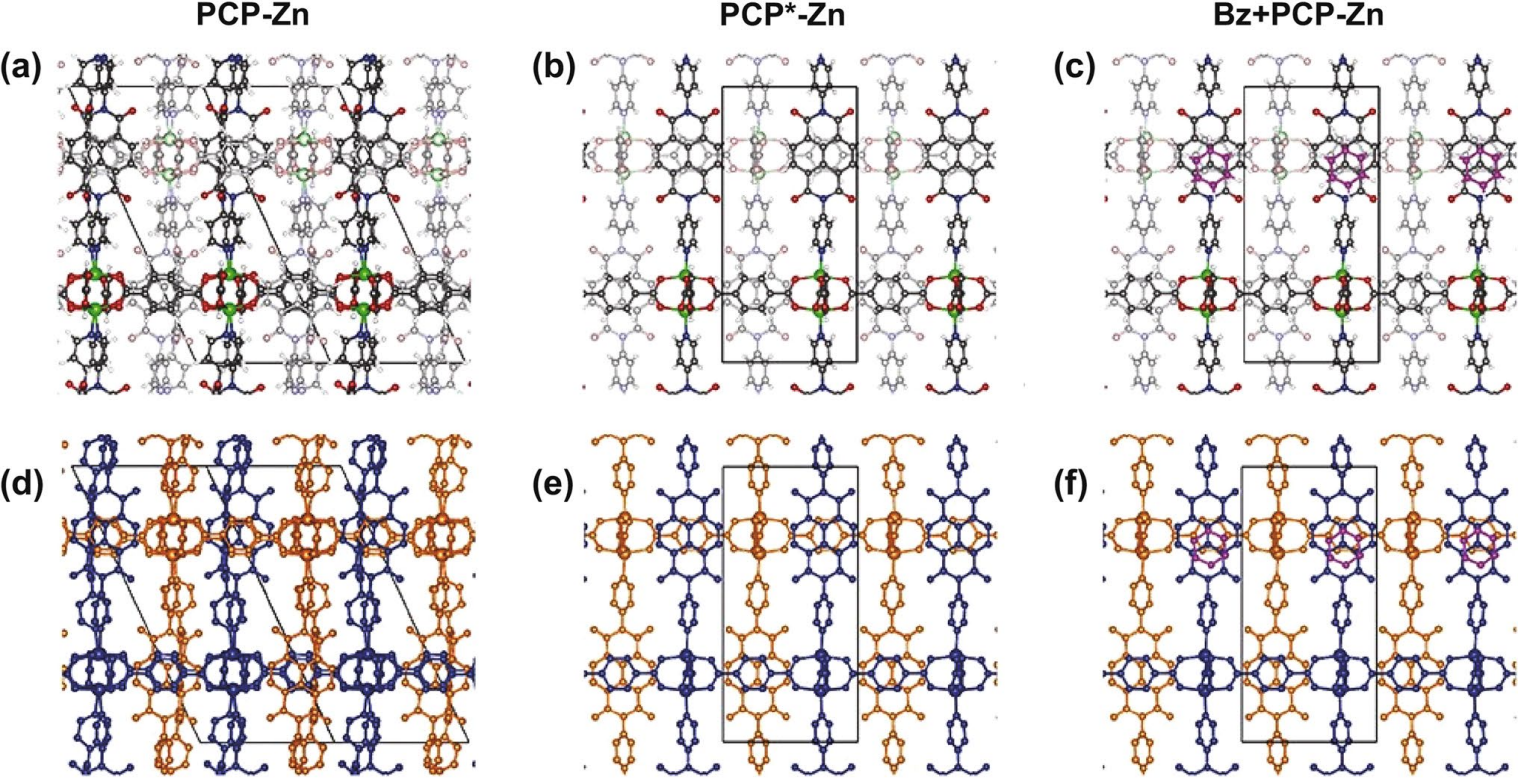
**a** PCP-Zn: Structure of pristine MOF before guest molecule introduction. **b** PCP*-Zn: Structure after guest-molecule-induced structural change, but without guest molecule. **c** Bz + PCP-Zn: Structure after adsorption of benzene in the MOF. **d–f** The same structures as a-c with subframeworks colored as blue (subframework 1) and orange (subframework 2) and the hydrogens removed for clarity. Reproduced with permission from Ref. [55]. Copyright 2019, American Chemical Society

**Fig. 20:**
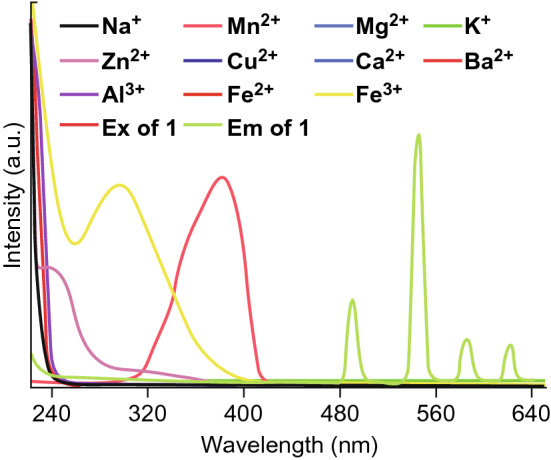
The emission (*λ*_ex_ = 380 nm) and excitation (*λ*_em_ = 545 nm) spectra of {[Tb(Cmdcp)(H_2_O)_3_]_2_(NO_3_)_2_·5H_2_O}_n_, as well as the absorption spectra of different metal ions. Reproduced with permission from Ref. [56]. Copyright 2019, Royal Society of Chemistry

Overall, to realize photoluminescence quenching or enhancement in MOFs for fabrication of practical luminescent sensors toward different kinds of target analytes, five possibilities are usually taken into consideration: (1) structural transitions of MOFs induced by target analyte; (2) ion-exchange or ligand-exchange induced by target analyte; (3) interactions between target analyte and the fluorophore in MOFs; (4) the overlap between the absorption spectrum and of the target analyte and the excitation spectrum of the MOFs; (5) the overlap between the absorption spectrum of the MOFs and the emission spectrum of the target analyte. The first possibility was demonstrated in Zn_2_(bdc)_2_(dpNDI) (PCP-Zn) [55]. Adsorption of benzene molecules into PCP-Zn would cause considerable transformation of the framework structure (Fig. 19), which significantly decreased the distance between HOMO_PCP*‑Zn_ and LUMO_PCP*‑Zn_ and localized the orbitals, increasing the oscillator strengths and rendering the pristine non-luminescent PCP-Zn strong photoluminescence. The luminescence enhancement made PCP-Zn a promising sensor for benzene detection. Examples for the second and third possibilities include Zn_2_(bpdc)_2_(bpee) [54] and Tb-SA [32], respectively, which have been discussed before. As for the fourth possibility, a typical example is {[Tb(Cmdcp)(H_2_O)_3_]_2_(NO_3_)_2_·5H_2_O}_n_ (H_3_CmdcpBr = N-carboxymethyl-(3,5-dicarboxyl)pyridinium bromide) as sensor toward Fe^3+^ [56]. As shown in Fig. 20, there was an obvious overlap between the adsorption spectrum of Fe^3+^ and the excitation spectrum of the MOF, which was not observed for other metal ions. The overlap revealed that Fe^3+^ would compete with the MOF to adsorb light energy, disabling the MOF to adsorb enough light to be excited and emit photoluminescence, leading to a quenching effect. The fifth possibility usually occurs between photoadsorptive MOFs and emissive target analyte, such as the aforementioned MOF-74(Zn)-en for TBBPA detection [53]. Notably, for the design of luminescent MOFs as sensors, Lewis acidic/basic active sites are often involved. In particular, Lewis basic sites have a strong chelating ability to Lewis acidic ions like Cu^2+^, Zn^2+^, La^2+^ [57], Fe^3+^ [58] and so forth, hence promoting the sensitivity of sensors toward these metal ions. For example, {[Cd_1.5_(DBPT)(DiPyDz)(H_2_O)]·3.5H_2_O}_n_ possess H_3_DBPT ligand that has open Lewis basic triazolyl groups, which can effectively bind to Fe^3+^ ions [58]. The limit of detection of this MOF toward Fe^3+^ can reach as low as 78 ppb, much lower than 112 ppb for [Cd(L)(pda)] [59].

#### Light Emitting

**Fig. 21:**
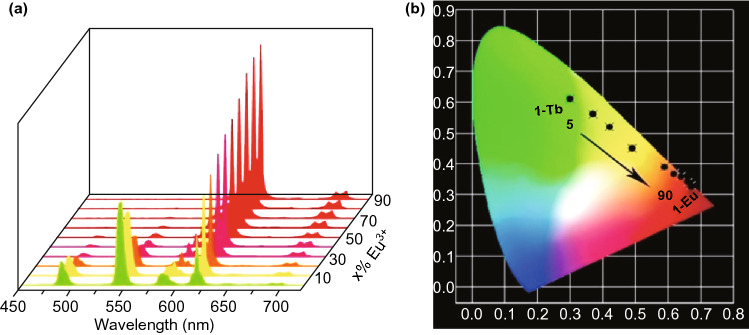
a Solid-state emission spectra of {[Eu_*x*_Tb_1−*x*_(L)(H_2_O)_2_]·5H_2_O}_n_ with different molar ratios of Eu^3+^ under excitation of 302 nm. **b** CIE chromaticity diagram of {[Eu_*x*_Tb_1−*x*_(L)(H_2_O)_2_]·5H_2_O}_n_. Reprinted with permission from Ref. [41]. Copyright 2019, American Chemical Society

More and more attention is being paid to the possible application of luminescent MOFs in lighting devices. Notably, based on the superior compositional tunability and diversity of MOFs, usually two or more emissive ions or molecules are involved in the construction of MOFs or guest@MOF hybrids to combine each emission color of each emissive component in order to obtain various high-quality colors. For example, a series of H_4_L^+^Cl^−^-based {[Eu_*x*_Tb_1−*x*_(L)(H_2_O)_2_]·5H_2_O}_n_ with both Eu^3+^ and Tb^3+^ ions were synthesized [41]. Dual emission of Eu^3+^ and Tb^3+^ was observed under excitation at 302 nm. As the molar ratio of Eu^3+^ increased from 5 to 90%, the luminescence intensity of Tb^3+^ at 544 nm gradually weakened and the luminescence intensity of Eu^3+^ at 617 nm gradually enhanced, as shown in Fig. 21a. This phenomenon was ascribed to enhanced energy transfer from Tb^3+^ to Eu^3+^ as molar ratio of Eu^3+^ increased, which gradually quenched the photoluminescence of Tb^3+^. Specifically, under irradiation at 254 nm, the luminescence colors of the MOF smoothly changed from yellow-green, yellow, orange, orange-red to red as the molar ratio of Eu^3+^ increased from 5 to 90% due to the synergy of Tb^3+^ and Eu^3+^ emissions, as shown in Fig. 21b. Multicolor emissions can be realized by modulating the molar ratio of different emissive components in one MOFs. In particular, based on three-primary colors theory, this dual emission mechanism has been widely applied in white-light-emitting field. The Commission International deI’Eclairage (CIE) color coordinates are used to assess the quality of white light emitted by various materials. The closer the CIE of MOFs is to the standardized coordinate for pure white light (0.3333, 0.3333), the higher the quality and purity of emitted white light are. And the different emissive components commonly used for synthesis of white-light-emitting MOFs with exceptional color quality and quantum yield are lanthanide metal ions, guest molecules and organic ligands.

**Fig. 22:**
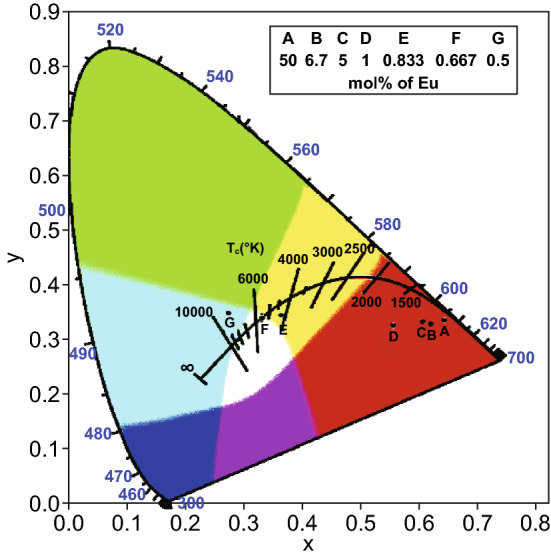
CIE chromaticity diagram of mixed Eu_*x*_Tb_1−*x*_-MOFs with different Eu ratios and excitation wavelengths. Reprinted with permission from Ref. [60]. Copyright 2019, Elsevier

Mixed-LnMOFs synthesized from two or more types of lanthanide metals are promising candidates for white light emission. A series of mixed-LnMOFs [(Eu_*x*_Tb_1−*x*_)_2_(TDC)_3_(CH_3_OH)_2_·(CH_3_OH)], abbreviated as Eu_*x*_Tb_1−*x*_-MOFs, were investigated with *x* = 0.5, 0.067, 0.05, 0.01, 0.00833, 0.00667, and 0.005 [60]. The CIE chromaticity diagram of MOFs with different Eu ratios is depicted in Fig. 22. It was found that only Eu_0.00667_Tb_0.99333_-MOF emitted white light under 350 nm excitation, with the CIE coordination of (0.3333, 0.3394) very close to that of pure white light. This research reveals that carefully tuning the ratio of different lanthanide metal ions in MOFs is a promising strategy to generate pure white light emission.

**Fig. 23:**
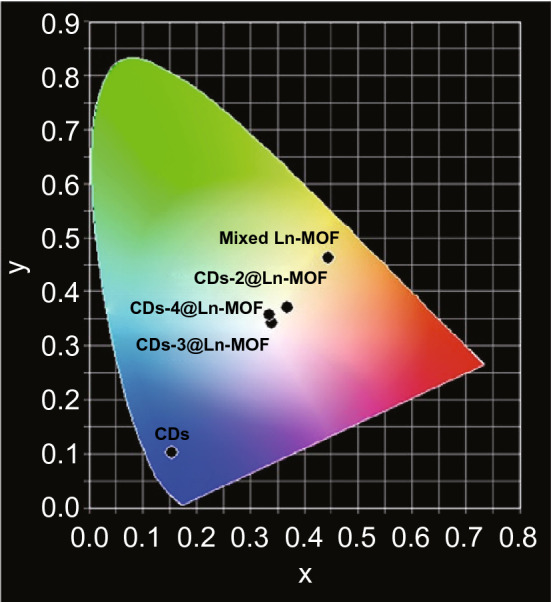
CIE chromatic diagram of CDs, mixed-LnMOF, and CDs@LnMOF hybrids. CDs-2, CDs-3, and CDs-4 refer to 2, 3, and 4 mL of CDs encapsulated into MOFs, respectively. Reprinted with permission from Ref. [63]. Copyright 2019, American Chemical Society

Dual emission for white light can be realized through introduction of luminescent guest molecules as well. The reported luminescent materials encapsulated into MOFs include iridium complex [61], fluorescent proteins (FPs) [62], carbon dots (CDs) [63], quantum dots (QDs), organic dyes and so on, as shown in Table 4. With optimal amount of guest molecules and excitation wavelengths, white light emission can be observed in the synthesized guest@MOF hybrids. As shown in Fig. 23, different amounts of blue-light-emitting carbon dots (CDs) were encapsulated into a mixed-LnMOF which emitted yellow luminescence and the final hybrids exhibited different luminescence colors upon irradiation [63]. For CDs-3@LnMOF with 3 mL CDs, different CIE coordinates were obtained under various wavelengths in range of 360–380 nm and the best CIE coordinate could reach up to (0.334, 0.334) when CDs-3@LnMOF was irradiated under 370 nm. This revealed that the CIE coordinates can be modulated through careful selections of guest molecule concentration and excitation wavelength. It should be noted that sometimes the luminescence bands of guest molecules will be influenced and changed upon encapsulation into MOFs. For example, R-phycoerythrin (R-PE) proteins were denatured after embedded into HSB-W1 framework, which inhibited their pristine orange luminescence at 578 nm and aroused new green (518 nm) and red (600, 647 nm) luminescence [62]. Synergy of emissions from R-PE and blue-light-emitting HSB-W1 finally resulted in high-quality white light emission with CIE of (0.33, 0.34).

**Table 4:** Photoluminescent MOFs for white-light emission

MOFs	Guest molecules	Excitation (nm)	CIE	References
HSB-W1	R-phycoerythrin (R-PE)	405	(0.33, 0.34)	[62]
Cd-MOF (CP1)	CdTe QDs	330	(0.33,0.32)	[110]
[Eu_1.22_Tb_0.78_(1,4-phda)_3_(H_2_O)](H_2_O)_2_	CDs-3	370	(0.334, 0.334)	[63]
[(CH_3_)_2_NH_2_]_15_[(Cd_2_Cl)_3_(TATPT)_4_]·12DMF·18H_2_O	[Ir(ppy)_2_(bpy)]^+^	370	(0.31, 0.33)	[61]
ZIF-8^2^	C-151	365	(0.16, 0.12)	[111]
	F		(0.26, 0.58)	
	RB		(0.57, 0.43)	
[Eu(MCTCA)_1.5_(H_2_O)_2_]·1.75H_2_O	H_4_TBAPy	350	(0.3482, 0.3301)	[112]
[Me_2_NH_2_][In(bptc)]	safranin O	380	(0.32, 0.33)	[113]
ZJU-28	Cou-6/R6G/R101	460	(0.36, 0.34)	[114]
[Eu_0.05_(H_2_O)_4_(pdc)]_4_SiMo_12_O_40_]·2H_2_O	Eu^3+^	295	(0.3425,0.2548)	[92]
	Eu^3+^/Tb^3+^		(0.3857,0.3377)	
[Zn_4_OL_2_·xDMF]_n_	DCM/C6	365	(0.32, 0.31)	[115]
Zr-MOF	CDs	365	(0.31, 0.34)	[116]

**Fig. 24:**
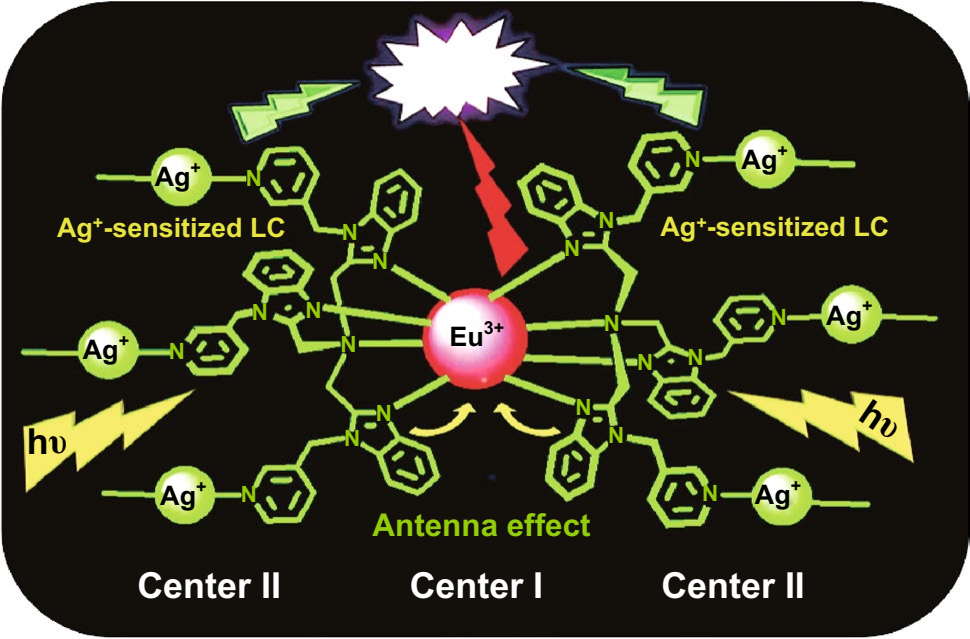
Dual-emitting pathways in the Ag-doped MOF generating white-light emission. Reprinted with permission from Ref. [64]. Copyright 2012, American Chemical Society

What is more, some LnMOFs with photoactive ligands can also generate dual emissions from Ln metal ions and organic ligands. In this case, the ligand-centered emission needs to be resensitized by dopant metal ions. For example, while the MOF [Eu(3-TPyMNTB)_2_](ClO_4_)_3_·2.5MeCN emitted characteristic red luminescence, the Ag-doped MOF [EuAg_3_(3-TPyMNTB)_2_(H_2_O)(MeCN)](ClO_4_)_6_·4MeCN directly emitted white light due to the ligand-centered emission of TPyMNTB resensitized by doped Ag^+^, as depicted in Fig. 24 [64].

One crucial challenge to light-emitting devices is the accompanying generated thermal energy. Under irradiation, a considerable amount of light energy absorbed by the device is converted to thermal energy, which diffuses into the surrounding environment and is hard to reuse, lowering the overall efficiency of the lighting device. What’s worse, the generated thermal energy will increase device temperature, which will probably decrease the device luminescence lifetime or even directly damage the device. Interestingly, an effective solution toward this challenge was proposed. Carbon quantum dot (CQD) and stearic acid (SA) molecules were simultaneously incorporated into Cr-MIL-101-NH_2_ to synthesize novel phase change materials (PCMs) [44]. As superior photoluminescent particles, CQD was utilized to render the composite light-emitting properties. Stearic acid functioned as thermal energy guest, which constantly adsorbed the generated heat in the process of photoluminescence, enabling thermal energy recycling and maintaining a relatively low temperature, hence improving luminescence efficiency and device lifetime. This PCMs system provides exciting improvement for lighting devices and is supposed to attract more and more attention.

#### Luminescent Thermometer

**Fig. 25:**
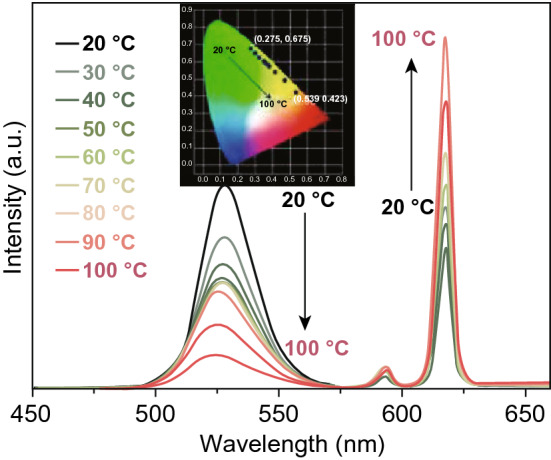
Temperature-dependent PL spectra of CsPbBr_3_@Eu-BTC in the temperature range of 20–100 °C excited at 339 nm (inset: the CIE (*x*, *y*) coordinate diagram of emission colors at various temperatures). Reprinted with permission from Ref. [65]. Copyright 2020, American Chemical Society

**Fig. 26:**
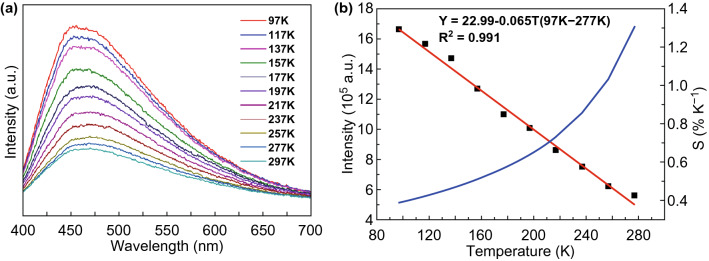
a PL emission spectra of C-QDs@UiO-66-(COOH)_2_ film in the temperature range of 97–297 K. **b** Emission intensity of the C-QDs@UiO-66-(COOH)_2_ film as a function of temperature (black squares, left axis) with the fitting curve (red line, R^2^ = 0.991) and the relative sensitivity curve (blue line, right axis). Reprinted with permission from Ref. [46]. Copyright 2018, American Chemical Society

Compared to traditional thermometers, such as liquid-filled thermometers, transistors, and thermocouples, which need direct physical contact with the tested environment, luminescent thermometers have attracted much attention due to their non-contact real-time temperature-sensing properties and can be applied in fast-moving samples and in strong magnetic or electronic situations. Excitingly, another promising application of photoluminescent MOFs is self-calibrating luminescent thermometer based on fluorescence intensity ratio (FIR) technique. In this case, dual emissions are required and the intensities of emissions at different wavelengths response to the temperature change differently. The intensity ratio of emissions at two wavelengths is the basis to measure temperature. One example is CsPbBr_3_@Eu-BTC which has been investigated in the temperature range of 20–100 °C and served as a reliable and stable thermometer with a high relative sensitivity (S_r_) of 3.9%/ °C at 20 °C and excellent temperature resolution of 0.004 °C [65]. As temperature increased, the photoluminescence at 528 nm from CsPbBr_3_ QDs got weaker, whereas the emission of Eu^3+^ at 618 nm became stronger, as shown in Fig. 25. Other luminescent MOFs with potential for temperature sensing based on FIR technique include the aforementioned [(Eu_0.0069_Tb_0.9931_)_2_(TDC)_3_(CH_3_OH)_2_·(CH_3_OH)] in the range of 288–353 K [60], Eu_0.0069_Tb_0.9931_-DMBDC (DMBDC = 2,5-dimethoxy-1,4-benzenedicarboxylate) in the range of 10–300 K [66], and so on. In addition, changes in the single-luminescence intensity at single wavelength of MOFs at different temperatures can also provide reference for temperature sensing. C-QDs@UiO-6-(COOH)_2_ [46] film can detect temperature change in the range of 97–297 K with the S_r_ value up to 1.3% K^−1^ at 297 K. Luminescence intensities of C-QDs@UiO-6-(COOH)_2_ at different temperatures are shown in Fig. 26a, and the relation between intensity and temperature is linear simulated in Fig. 26b.

#### Optical Information Protection

**Fig. 27:**
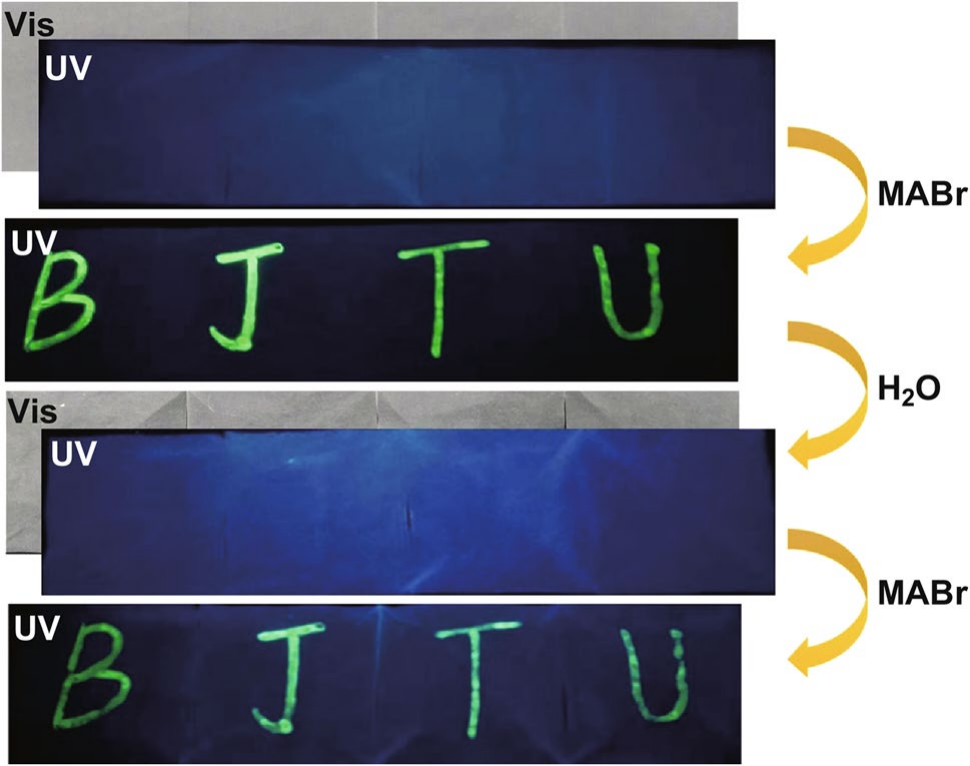
Reversible fluorescence switching of the BJTU pattern written on the paper at different stages under ambient and 365 nm UV light. Reprinted with permission from Ref. [43]. Copyright 2019, Elsevier

Based on the quenching and recovery of luminescence, the potential of MOFs for information encryption and decryption has also been investigated. MAPbBr_3_@UiO-66, synthesized by simply encapsulating the conventional luminescent MAPbBr_3_ perovskite into the MOF UiO-66, was such stable material that it was used for information protection and anti-counterfeiting because MAPbBr_3_ could be converted into PbBr_2_ by water and would recover when treated with MABr solution, as shown in Fig. 27 [43]. The single and bimetallic MOFs [Eu_*x*_Tb_2−*x*_(1,4-phda)_3_(H_2_O)](H_2_O)_2_ (*x* = 0, 0.73, 1.22, 1.57, 1.94, and 2) have also been demonstrated to serve as luminescent security inks [63]. In this case, special information storage boxes were utilized, which were transparent under daylight but could be excited to emit luminescence under UV light. Letters were written on box by MOF ink that emitted the same luminescence color as the box. Addition of styrene could quench the luminescence of the MOF due to energy transfer from the MOF to styrene, enabling decoding of the letters under UV light. Besides, styrene could easily evaporate in air, making it possible to erase and rewrite the letters.

## Deposition of MOF Thin Films

For device integration, it is required that MOFs possess enough physical contacts with other materials and to meet this requirement, MOFs are often prepared in forms of thin films. It is of great significance to master how to fabricate high-quality MOF thin films with precise control over the thickness, morphology, density, crystallinity, roughness, and orientation, which determine the device performance of MOFs [67]. For conductive MOFs, it has been demonstrated that thickness [68] and orientation [69] of thin films can affect the electrical conductivity. For photoluminescent MOFs, thin films possess advantages over powders, such as more binding sites for analyte molecules or ions, easy separation from solutions, less crystal defects and so forth. Many methods have been developed for deposition of MOF thin films, some of which have been demonstrated to exhibit flexibility toward various MOFs.

### Electrochemical Deposition

**Fig. 28:**
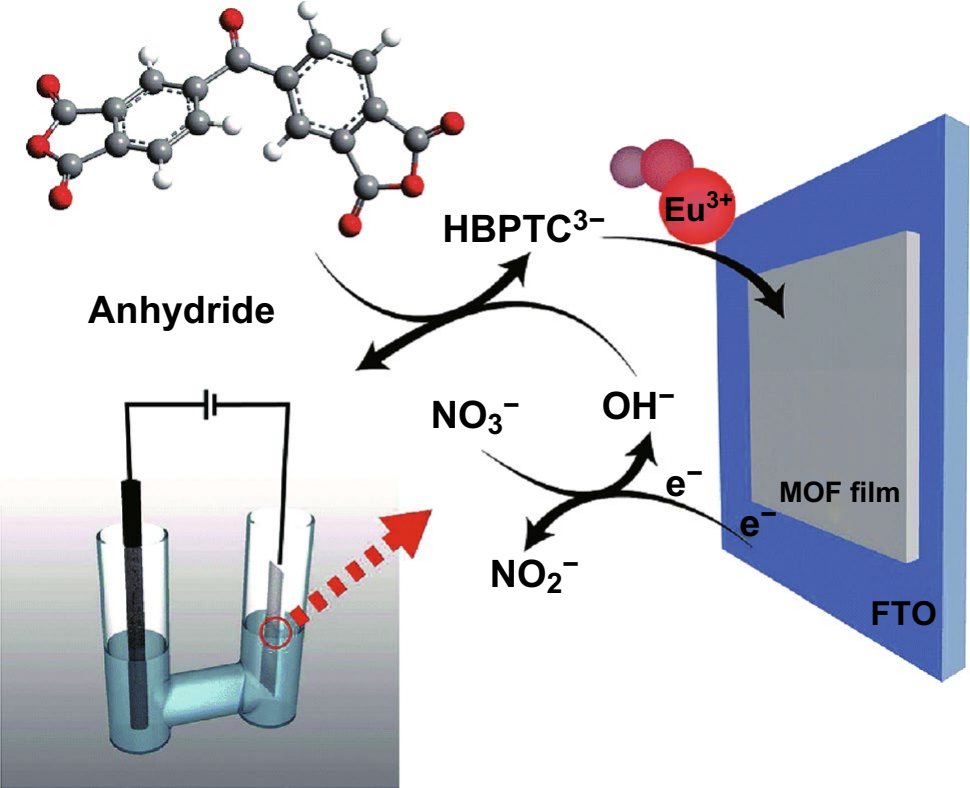
Cathodic deposition of Eu-HBPTC thin film. Reprinted with permission from Ref. [70]. Copyright 2014, Royal Society of Chemistry

Electrochemical deposition, including cathodic and anodic deposition, is a rapid method to fabricate MOF thin films and allows for mechanical and electrical contact between the MOF and substrate. For cathodic deposition, precursor metal ions and ligands are both required in electrolyte and the MOF thin film deposits on the surface of cathode. For instance, as shown in Fig. 28, with a graphite rod as the anode and the fluorine-doped tin oxide (FTO) conductive glass as the cathode, the Eu-HBPTC thin film appeared on the cathode when the two electrodes were immersed into the mixed solution of benzophe-none-3,30,4,40-tetracarboxylic dianhydride (BTDA), DMF and Eu(NO_3_)_3_·6H_2_O and a constant current was applied [70]. The thus synthesized Eu-HBPTC thin film presented the similar emission spectra to Eu^3+^ ions and could be used as a highly selective sensor for carbonate in aqueous solution even with the CO_3_^2−^ concentration down to 10^−4^ M. Similarly, this method has succeeded in fabrication of the aforementioned MOF terbium-succinate (Tb-SA) thin film as a sensor for Cu^2+^ [32]. Furthermore, using the same method, white-light-emitting thin films of LnCPs, formulated as [Ln_6_(HMA)_6_(H_2_O)_16_]·17H_2_O (HMA-Ln, Ln = Eu^3+^, Gd^3+^, Tb^3+^; H_3_HMA = hemimellitic acid), were fabricated and exhibited satisfactory CIE coordinates reaching (0.33, 0.34) [71].

However, for anodic deposition, the MOF thin film deposits on the anode and the electrolyte only contains the precursor organic ligands because metal ions for MOF construction come from the anode. A series of MOFs were deposited on indium tin oxide (ITO) glass previously coated by corresponding metallic films through anodic deposition, which proved to be a promising strategy for integration of MOFs with electronic devices, and attempts of involving more conductive MOFs in this processing are under way [72].

**Fig. 29:**
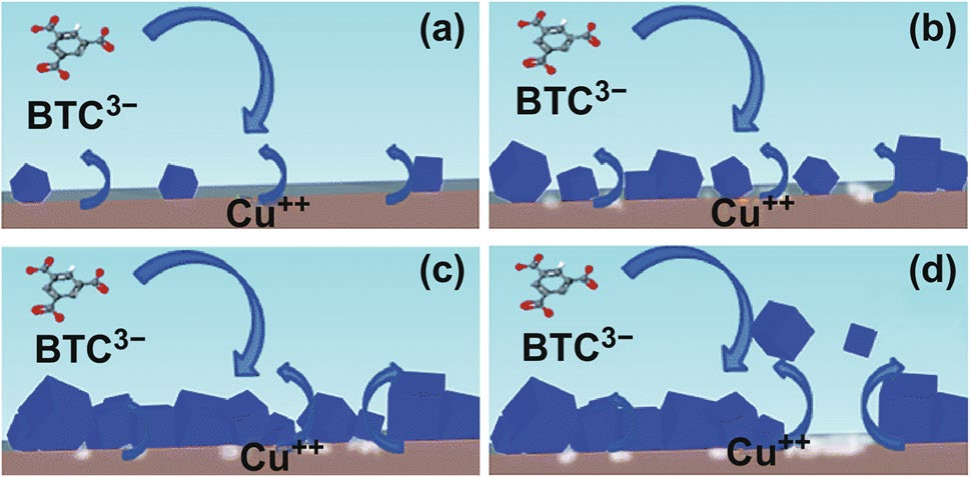
Four phases of anodic deposition: **a** initial nucleation, **b** growth of MOF islands, **c** intergrowth, and **d** crystal detachment. Reprinted with permission from Ref. [73]. Copyright 2016, Royal Society of Chemistry

The difference between anodic deposition and cathodic deposition was investigated by comparing the anodic deposition of Cu-INA, Cu-INA(Cl), and Cu-INA(F) with the cathodic deposition of HKUST-1 [73]. It was demonstrated that the anodic deposition consists of four phases: initial nucleation, growth of MOF islands, intergrowth, and crystal detachment, as shown in Fig. 29. A lag time is needed for anodic deposition depending on the applied current and the metal-ion concentration threshold for MOF nucleation, while cathodic deposition can start at potentials less cathodic. However, anodic deposition facilitates better manipulation of the film characteristics like film thickness, crystal size, and morphology by varying synthesis parameters like voltage and current density, concentrations of ligands and conduction salt and temperature [72]. Though anodic deposition has been widely adopted to fabricate films with excellent electrocatalytic and proton-conductive properties [74, 75], investigations on MOF films with photophysical properties as luminescent sensors or photoconductive electrodes fabricated through anodic deposition are still limited.

### Electrophoretic Deposition

Electrophoretic deposition is based on the fact that the suspended MOFs possess a surface charge. By immersing two identical conductive electrodes into the colloidal MOF suspension and applying a fixed voltage between the two electrodes, the MOF particles will move toward the oppositely charged electrode driven by the electric field and hence form a thin film. Interestingly, this method enables MOF particles to deposit on predefined positions and form micropatterned films. Take the fabrication of NU-1000 thin films on FTO for example: bare FTO platform was firstly modified with an insulating photoresist layer, and then photolithography was applied to create certain micropatterns of the photoresist layer; with NU-1000 deposited only on the exposed sections of FTO through electrophoretic deposition, followed by the removal of photoresist materials by immersing the platform in acetone, a micropatterned NU-1000 thin film was thus formed [76].

Through electrophoretic deposition, continuous and dense thin films of a series of photoluminescent LnMOFs were successfully fabricated on unmodified low-cost substrates including zinc plate, ITO and FTO glasses, rapidly in 5 min [77]. In particular, as-synthesized Tb-BTC films exhibited exceptional performances in the detection of nitrobenzene (NB) and Cr^3+^ in solution and trinitrotoluene (TNT) and NB in gas phases. In addition, for the sake of ratiometric temperature-sensing thin films, two dual-emitting Ln@UiO-66-Hybrid MOFs, with lanthanide metals and luminescent ligand integrated in a UiO-66-type structure, were deposited on FTO substrates through electrophoretic deposition where the charges from uncoordinated carboxylic groups played a critical role [78]. The thus synthesized Tb@UiO-66-Hybrid film was able to measure temperatures in range of 303–353 K with a relative sensitivity of 2.76% K^−1^, while the temperature range and relative sensitivity for Eu@UiO-66-Hybrid film were 303–403 K and 4.26% K^−1^, respectively. Later on, the same group used the same electrophoretic deposition methodology to fabricate C-QDs@UiO-66-(COOH)_2_ composite thin film as a temperature sensor in range of 97–297 K with a relative sensitivity of up to 1.3% K^−1^ [46]. The film exhibited better temperature-sensing performances than non-film-state C-QDs@UiO-66-(COOH)_2_ composites, which to some extent corroborated the more excellent capacities of thin films.

### Layer-by-Layer Assembly

Layer-by-layer assembled method relies on the in situ growth of MOFs on different substrates. In general, the process includes repeating growth cycles of stepwise immersion of the substrate into solution of metal ions and then solution of organic ligands. The substrate is usually modified with a self-assembled monolayer (SAM) such as an organic linking molecular or metal-oxide film, to facilitate the strong adhesion of MOFs to the substrate during crystal growth and better control the interface of the bottom substrate and the MOF films. It has been validated that the SAM surface can affect the nucleation and further influence the crystal growth [79]. The film thickness can be well controlled by the number of growth cycles.

**Fig. 30:**
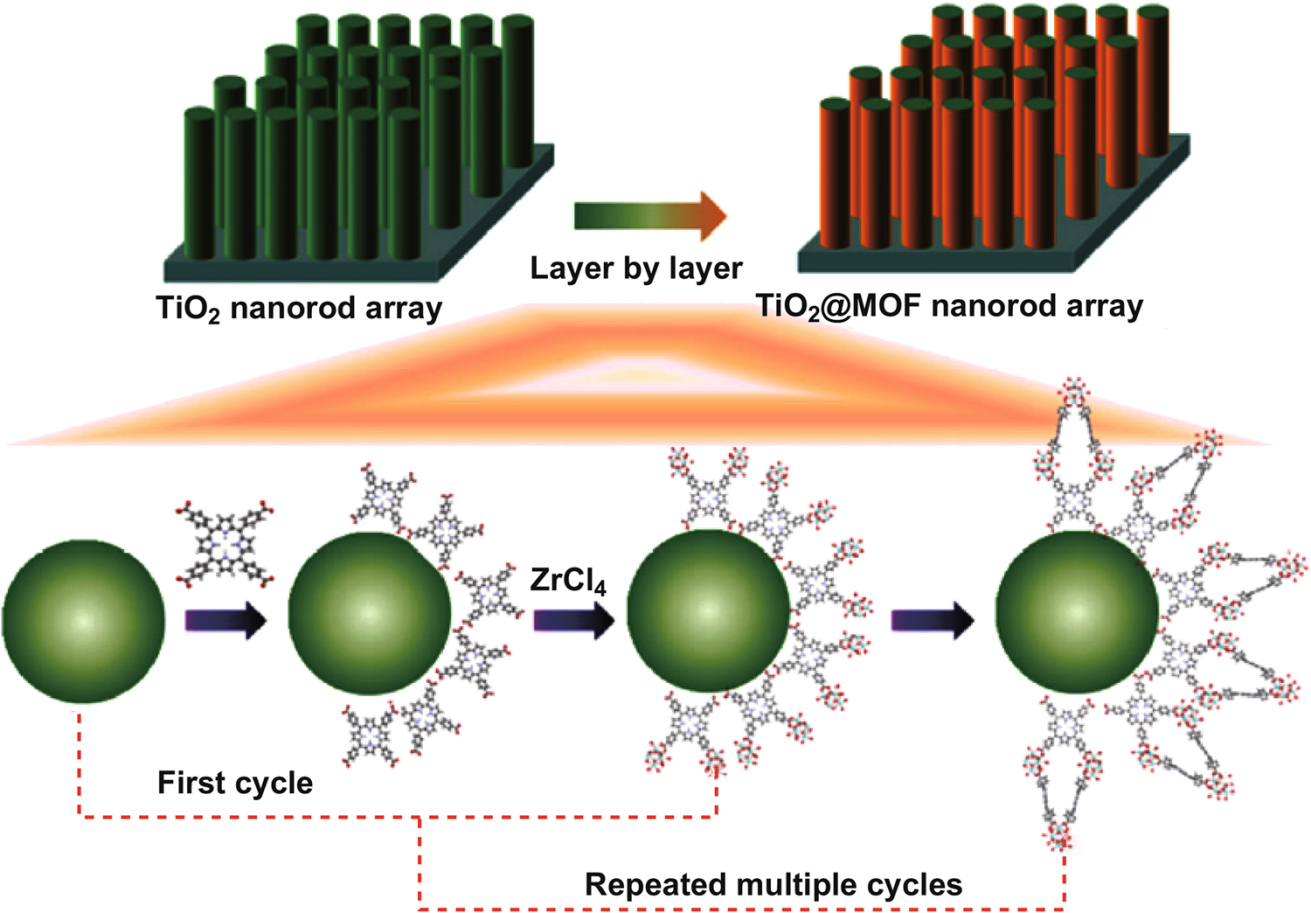
Synthesis of TiO_2_@MOF nanorod array photoanode through layer-by-layer method. Reprinted with permission from Ref. [28]. Copyright 2018, Springer Nature

A p–n heterojunction photoanode for solar water splitting was fabricated by coating a porphyrin-based MOF PCN-225 layer on a vertically aligned TiO_2_ nanorod array through layer-by-layer self-assembly [28]. The specific processing is shown in Fig. 30. The TiO_2_ nanorod arrays were alternately soaked into a 0.5 mM TCPP in ethanol solution and into a 2 mM ZrCl_4_ in ethanol solution at 40 °C with intervals set as 10 min. The above treatments were repeated for 5 cycles to obtain PCN-225 films with ideal thickness, and subsequently, the TiO_2_@MOF samples were heated at 150 °C under an N_2_ gas environment to strengthen the contact between the MOF and TiO_2_. The thus synthesized TiO_2_@Co-MOF photoanode presented a photocurrent density of up to 2.93 mA cm^−2^ at 1.23 V (vs. RHE).

Interestingly, through layer-by-layer assembly method, Eu-SURMOF was deposited on top of Tb-SURMOF to form a hetero-multilayer architecture, which suppressed direct energy transfer from Tb(III) to Eu(III) and thereby made the modulation of the emission color easier [80]. An Eu-NDC@HPNA thin film was also fabricated through this method and served as a luminescent sensor for formaldehyde, an illegal preservative in aquatic product, indicating that luminescent MOFs could play a significant role in food industry and our health [81].

While layer-by-layer assembly method has many advantages such as well-controlled thickness and mild reaction condition at room temperature, there are still some disadvantages like tedious repeating operations, long reaction times, and so on. Therefore, some improved methods have been developed. A promising alternative method is to use the metal oxide itself as a template for MOF growth by sequential exposure to the metal cation and then the organic linker. With aluminum-doped zinc oxide (AZO) as a seed layer, copper benzene-1,3,5-tricarboxylate (Cu-BTC) MOF growth occurs rapidly only on the AZO surface and it is found that Cu-BTC morphology can be optimized through careful choice of the Cu salt, solvent system, and pH [82]. It was also found that zeolite imidazolate framework-8 (ZIF-8) can directly assemble on gold surfaces when modified by cysteamine in colloidal suspensions, without the need to pretreat the substrate with SAM [83]. One of the challenges that block wide applications of layer-by-layer assembly in fabrication of electronic or optoelectronic devices lies in that it commonly relies on insulating SAMs to control the thin-film orientation, which could impede charge transportation. Inspired by these investigations, more convenient preparations of MOF thin films for high-performance photoelectronic and photoluminescent devices through better improved layer-by-layer assembly method should be included in future researches.

### Solvothermal Deposition

The solvothermal growth of MOF films is a facile, efficient, and low-cost deposition method and thus has been widely adopted. Upon heating, MOFs growth occurs rapidly on the substrate surface. In general, this method allows for direct and oriented deposition of MOF particles on semiconducting metal-oxide-coated electrodes, which thereby makes it more attractive for production of electronic and optoelectronic devices.

**Fig. 31:**
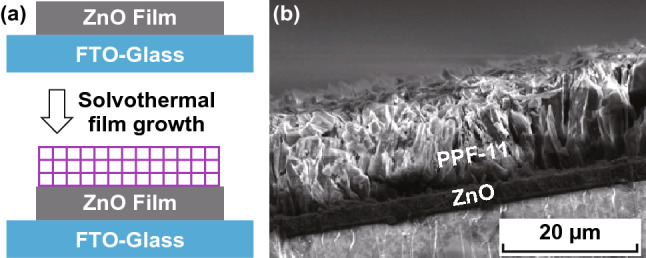
a Schematic diagram of the solvothermal growth of PPF-11 film. **b** Cross-sectional-SEM images of solvothermally grown 10 μm thick PPF-11 film on ZnO layer. Reprinted with permission from Ref. [84]. Copyright 2019, American Chemical Society

Under solvothermal conditions, pillared porphyrin framework-11 (PPF-11) featuring Zn-tetrakis(4-carboxyphenyl)porphyrin (ZnTCPP) and 2,2′-dimethyl-4,4′bipyridine was deposited on ZnO-coated FTO electrodes to form precisely [100]-oriented films, as shown in Fig. 31 [84]. DMF/EtOH solutions of Zn(NO_3_)_2_·6H_2_O, TCPP, DMBPY and 1 M HNO_3_/EtOH were heated at 80 °C for 2 h, followed by the immersion of annealed ZnO–FTO slides into the above precursor solutions at upright positions at 80 °C for 30 min, which led to spontaneous formation of uniform crystalline films. Solar cells based on the as-synthesized PPF-11/ZnO–FTO photoanode exhibited superior photovoltaic response with power conversion efficiency up to 0.86%, which was significantly linked to the covalent attachment to ZnO surface and [100] orientation of PPF-11 films.

Solvothermal deposition has also been utilized to generate MOF thin films as efficient luminescent sensors [85–87]. MOF-5 was deposited on ZnO-coated FTO substrate solvothermally, followed by postsynthetic introduction of Tb^3+^ [85]. Tb(III)@MOF-5/ZnO was demonstrated to detect acetone molecules with high selectivity due to the luminescence response of Tb^3+^ ions. In this study, the failure of MOF-5 deposition on bare FTO revealed that ZnO coating was necessary for MOF-5 growth. Similarly, MIL-124@Eu^3+^ film was deposited on porous ɑ-Al_2_O_3_ plate as ammonia sensor with the limit of detection of 26.2 ppm [87].

### Liquid–Liquid Interfacial Method

Liquid–liquid interfacial method is another facile and rapid method to obtain MOF thin films. Typically, it starts with the preparation of two immiscible liquid systems, which dissolve metal-ion salts and organic ligands, respectively. Then, by simply layering one of the liquid systems onto another, the MOF thin film appears at the liquid–liquid interface and can be observed through eyes [68, 88]. Further improvement of this method combines the spray technique by spraying the atomized solution of metal ions onto solutions of ligands [89, 90]. Since thin films form at liquid–liquid interface and can be easily separated from the liquid, this method is promising for fabricating free-standing MOF thin films without substrates. The main challenge of this method lies in the careful selection of immiscible solvents to dissolve metal ions and ligands, respectively. However, with its facile, convenient and time-saving advantages, liquid–liquid interfacial method holds a bright prospect for integration of MOF thin films with optical devices and should attract more attention.

### Ultrasonic Spray Deposition

**Fig. 32:**
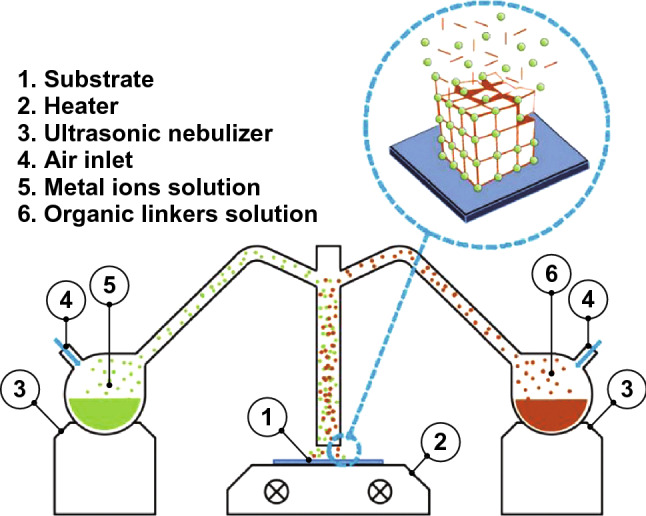
Scheme of the ultrasonic spray deposition system. Reprinted with permission from Ref. [91] Copyright 2019, Elsevier

Ultrasonic spray deposition is a novel strategy for MOF thin-film fabrication. The process of this technique is shown in Fig. 32. Two precursor solutions of metal ions and organic ligands are held in two separated ultrasonic nebulizers to generate corresponding ultrafine mists, which are then transported through a gas flux and mixed on the heated substrate surface, where solvents evaporate and MOFs crystalize to form matrix-free thin films. Following the above processing, Tb_2_(BDC)_3_ (BDC = 1,4-benzenedicarboxylate) MOF films were deposited on various substrates and exhibited photoluminescent properties [91]. It was revealed that the temperature of the substrate played a crucial role in the structures, morphologies, and luminescence properties of the as-synthesized films and low temperatures tended to generate films with higher luminescence intensities. As a time-saving, low-cost, and scalable new route for fabrication of luminescent MOF films, ultrasonic spray deposition can be considered a breakthrough for integration of MOFs in future optical devices.

### Other Methods

Apart from the aforementioned methods, many other methods have also been developed to fabricate various MOF thin films with excellent photophysical properties. Spin coating, for example, has proved to be successful in the fabrication of white-light-emitting Ln^3+^-functionalized [La(H_2_O)_4_(pdc)]_4_[SiMo_12_O_40_]·2H_2_O thin films [92] as well as white-light-emitting Sm^3+^@NENU-5 and Eu^3+^/Tb^3+^@NENU-5 thin films [93]. Besides, Langmuir–Blodgett method was utilized to fabricate semiconducting Cu-PPFs thin films for photoelectric conversion [94]. It should be noted that films of the same MOF fabricated through different methods usually differ in morphologies, surface coverage rate, and hence device performances. A research pointed out that luminescent MOF-76(Tb) films fabricated through hydrothermal, microwave-assisted, and layer-by-layer methods presented pillar-like, sedimentary-rock-like, and needle-like crystal morphology, respectively, and layer-by-layer method achieved the highest surface coverage rate due to promoted metal-ion anchoring [95]. Sometimes, more than one method will be adopted to combine advantages of each method to fabricate better MOF films.

## Conclusion and Outlook

In summary, conductive MOFs with photoconductive and photoluminescent properties have been widely investigated. Compared to conventional energy such as fossil fuels and natural gas that is limited in nature and contaminates our environment, light energy possesses superior advantages such as renewability and eco-friendliness. For effective utilization of light energy, many novel materials have been developed and MOFs with excellent photophysical properties provide another possibility to this end. Photoconductive MOFs are promising materials for solar cells and water splitting with superior light adsorption capacity, high stability, low cost, and many other advantages. Photoluminescent MOFs exhibit a bright prospect in many interesting fields such as luminescent analyte sensing, temperature sensing, light emitting, and optical information protection. In addition, thin films based on these photoconductive and photoluminescent MOFs have been reported, making it possible to integrate these MOFs with practical devices.

However, still there are challenges for further development of these MOFs and more efforts should be done in many future works. For example, to date most of photoconductive MOFs actually exhibit relatively low electrical conductivity despite their superior light adsorption capacity, which to some extent restrains their application in solar cells. Attempts should be continued to synthesize MOFs with higher electrical conductivity and higher photoelectric conversion efficiency. Also, most of the reported methods to fabricate MOF thin films are only applicable to some specific MOFs, and therefore, it is of great significance to search for more facile and more flexible methods for MOF thin-film fabrication. It is believed that these advances will definitely extend the applications of MOFs to electronic and optoelectronic devices and probably arise impactful innovation in the field of materials.
